# Endogenous Engineering Reprograms Extracellular Vesicles for Enhanced Therapeutic Function

**DOI:** 10.1002/advs.202524231

**Published:** 2026-05-25

**Authors:** Jinghui Wang, Wenxuan Zhao, Xiaoming Hu, Yishu Wang, Peidong Yang, Diwei Zheng, Yugang Wang, Guanghui Ma, Min Shi, Wei Wei, Shuang Wang

**Affiliations:** ^1^ Department of Gastroenterology Tongren Hospital Shanghai Jiao Tong University School of Medicine Shanghai P. R. China; ^2^ State Key Laboratory of Biopharmaceutical Preparation and Delivery Institute of Process Engineering Chinese Academy of Sciences Beijing P. R. China; ^3^ School of Chemical Engineering University of Chinese Academy of Sciences Beijing P. R. China; ^4^ Department of Breast Surgery Affiliated Quanzhou First Hospital of Fujian Medical University Quanzhou Fujian P. R. China

**Keywords:** endogenous engineering, extracellular vesicles, nucleic acid, protein cargo, therapeutic applications

## Abstract

Extracellular vesicles (EVs) have emerged as versatile biological carriers capable of transporting diverse therapeutic cargos. Their endogenous biogenesis pathways provide unique opportunities to regulate cargo selection through both environmental modulation and genetic programming. This review outlines how EV‐producing cells can be reprogrammed to load functional proteins and nucleic acids by combining environmental cues with genetic modifications that leverage intrinsic sorting machinery and engineered molecular interactions. For protein cargo, we outline strategies that reshape EV composition through physiological stimuli, as well as genetically encoded systems designed to recruit proteins via scaffold fusion, peptide tags, or fusion‐independent mechanisms. For nucleic acid cargo, we highlight approaches that leverage environment‐driven alterations in RNA abundance, together with targeted loading methods that rely on scaffold‐RNA‐binding proteins (RBPs) fusion constructs, natural sorting motifs, and sorting‐related RBPs to enrich miRNAs, mRNAs, and ribonucleoprotein complexes. We further summarize recent therapeutic applications of these endogenous engineering strategies in cardiovascular, hepatic, neurological diseases, and cancer. Finally, we discuss future directions, including high‐throughput discovery of sorting elements, scalable biomanufacturing, and improved standardization, which together will advance the development of programmable and clinically translatable EVs‐based therapeutics.

## Graphical abstract

This review explains how Extracellular vesicles‐producing cells can be endogenously engineered to load therapeutic proteins and nucleic acids. We summarize physiological and genetic strategies that harness native sorting pathways for selective cargo loading. We also present representative disease applications of these approaches in cardiovascular, liver, and neurological diseases, as well as cancer.

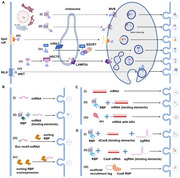

## 1 Introduction

Extracellular vesicles (EVs) are nanoscale, membrane‐enclosed particles released by nearly all prokaryotic and eukaryotic cells, and are broadly categorized based on their biogenesis pathways and physical characteristics. These include small EVs (often historically termed “exosomes”), which are formed as intraluminal vesicles (ILVs) within multivesicular bodies (MVBs), driven by endosomal sorting complex required for transport (ESCRT) and other cellular mechanisms, and released upon MVB‐plasma membrane fusion. In contrast, large EVs (commonly referred to as “microvesicles” or “ectosomes”) arise from the direct outward budding and fission of the plasma membrane, a process regulated by cytoskeletal rearrangement and calcium influx [1]. Additionally, other EV subtypes, like apoptotic bodies, are released during programmed cell death [2]. While this review uses the inclusive term “EVs” throughout, the strategies and applications discussed herein predominantly concern small EVs (i.e., exosomes), owing to their nanoscale size, relatively clear biogenesis mechanism, and broad use as delivery platforms in therapeutic research.

EVs are important mediators of intercellular communication and can transport proteins, lipids, metabolites, and nucleic acids to regulate various biological processes [3]. Their molecular contents often reflect the physiological or pathological state of the parental cells and may change in response to environmental stimuli [4]. Despite their potential, native EVs face therapeutic bottlenecks, including low endogenous cargo concentrations, insufficient targeting specificity, rapid clearance by the mononuclear phagocyte system, and poor functional delivery to recipient cells. To address these issues, different engineering strategies have been developed to improve cargo loading, enhance targeting capability, prolong circulation time, and promote intracellular delivery. These modifications may help convert native EVs into more efficient and controllable therapeutic delivery platforms for complex diseases.

EVs can be engineered for therapeutic applications through exogenous or endogenous strategies. Exogenous methods, such as co‐incubation, electroporation, sonication, freeze–thaw cycling, or surface conjugation, are relatively straightforward. However, these methods may damage EV membrane integrity and often show limited loading efficiency and cargo stability, especially for large or complex biomacromolecules [5]. In contrast, endogenous cell bioengineering harnesses the cell's intrinsic biosynthetic and sorting machinery for cargo incorporation, preserving both vesicle and cargo integrity while enabling stable and efficient loading of complex biomacromolecules. In this review, we focus on the more promising endogenous loading strategies for loading proteins and nucleic acid cargo. Environmental stimuli can reshape the expression profiles of parental cells and alter the protein and RNA composition of their secreted EVs. In addition, genetic engineering enables the selective enrichment of therapeutic proteins or nucleic acids in EVs by utilizing the cellular pathways responsible for MVB formation and plasma membrane remodeling. This strategy provides a programmable and efficient means of cargo loading, and forms the foundation for next‐generation EV‐based therapeutics.

In this review, we first summarize the molecular mechanisms underlying EV biogenesis and then highlight endogenous engineering strategies for generating therapeutic EVs. For protein cargos, we introduce approaches that utilize environmental cues to reshape EV composition, as well as genetically encoded systems that guide proteins into EVs through scaffold fusion, recruitment tags, or controlled intraluminal release. We also discuss fusion‐independent methods that rely on natural trafficking chaperones or fusogenic proteins to load unmodified proteins into EVs. For nucleic acid cargos, we outline strategies that modulate endogenous RNA profiles through environmental stimulation, along with targeted loading approaches that make use of natural sorting motifs, cognate RBPs, and engineered RNA‐protein recruitment systems to enrich miRNAs, mRNAs, and ribonucleoprotein (RNP) complexes in EVs. We also highlight representative therapeutic studies that demonstrate how these engineering principles can be applied across diverse biological contexts, including cardiovascular, hepatic, neurological diseases, and cancer. Finally, we discuss current challenges and future directions that may shape the next generation of EV engineering strategies.

## 2 Natural Mechanisms of Molecule Sorting in Extracellular Vesicles

**FIGURE 1:**
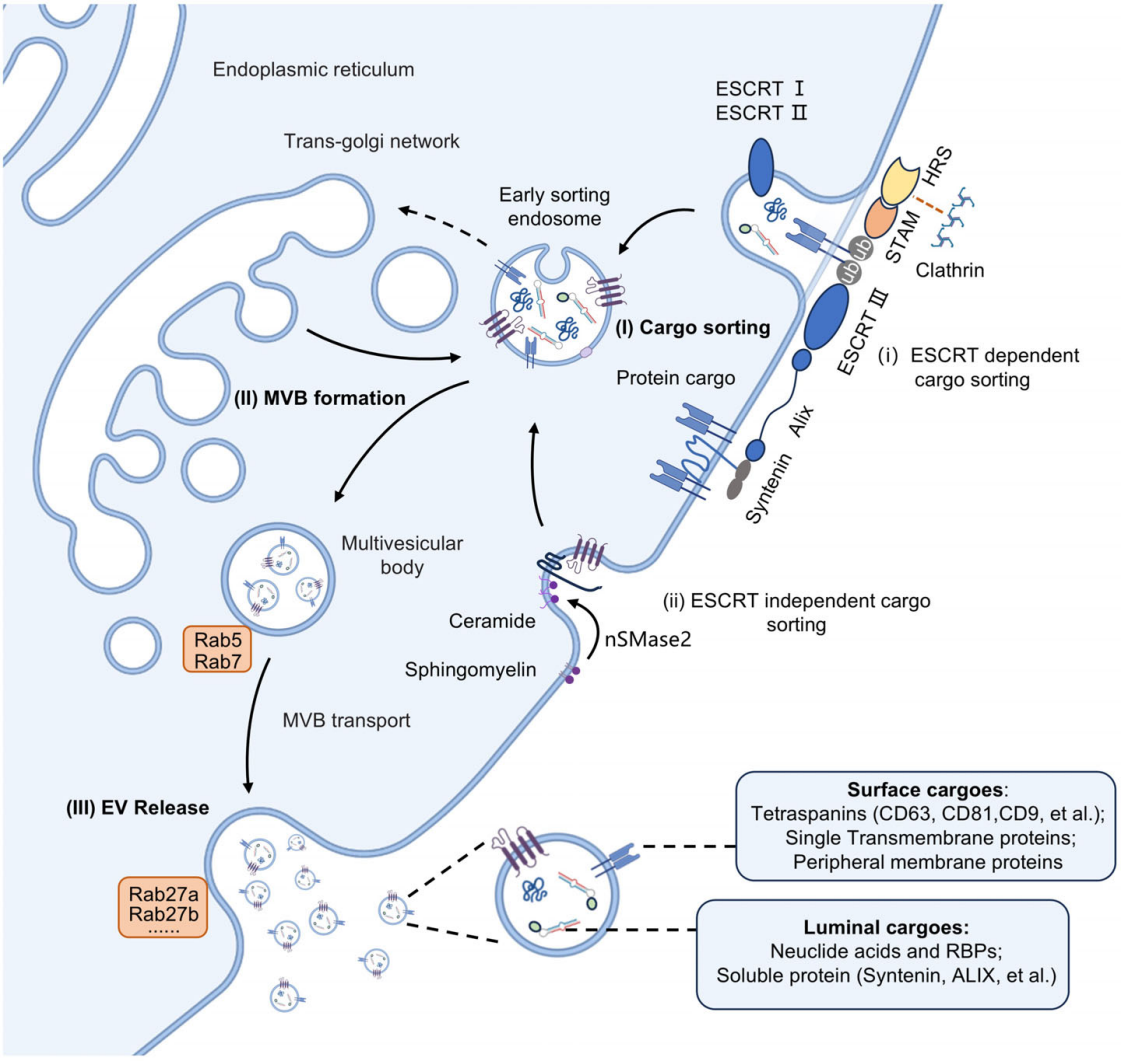
Schematic illustration of the molecular mechanisms governing extracellular vesicle (EV) biogenesis and release. (I) Cargo sorting and membrane remodeling through the endosomal sorting complex required for transport (ESCRT)‐dependent machinery and the ESCRT‐independent pathway [47]. (i) ESCRT‐dependent cargo sorting. Recruitment of ubiquitinated protein cargoes by clathrin, HRS, and STAM, followed by ESCRT‐I, II, and III‐mediated vesicle budding, supported by the Syntenin‐Alix axis. (ii) ESCRT independent cargo sorting. Membrane remodeling and cargo clustering driven by nSMase2‐mediated ceramide generation and the scaffolding functions of tetraspanin‐enriched microdomains. (II) multivesicular bodies (MVBs) formation. The transition from early sorting endosomes to MVBsvia a Rab5‐to‐Rab7 switch. (III) EV release. Rab27a/Rab27b‐regulated fusion of MVBs with the plasma membrane, culminating in the secretion of EVs. The released EVs are depicted with their characteristic cargo topology: transmembrane proteins are displayed on the EV surface, while cytosolic proteins and nucleic acids are encapsulated within the EV lumen.

Current insights into the mechanisms of EV biogenesis are predominantly established based on endosome‐derived vesicles. This biogenic pathway represents a multistep process that integrates membrane remodeling, intracellular trafficking, and cargo selection. It typically proceeds through three coordinated stages: cargo sorting, mediated by mechanisms such as the ESCRT complex or nSMase2‐dependent ceramide generation; MVB formation, which involves the transition from early endosomes via a Rab5‐to‐Rab7 switch; and EV release, culminating in the Rab27a/Rab27b‐regulated fusion of MVBs with the plasma membrane (Figure 1) [6]. The functional diversity of EVs arises from the heterogeneity of their cargo. Although cells contain thousands of biomolecules, including proteins, nucleic acids, and lipids, not all intracellular molecules are incorporated into these vesicles. Parental cells utilize a suite of sophisticated molecular machineries to achieve the selective sorting and loading of specific cargo, a process that ultimately defines the functional identity of the EVs. Their formation involves several molecular machineries, including the ESCRT complex, tetraspanins, sphingomyelinase‐driven lipid remodeling, phospholipid redistribution, and actin cytoskeleton depolymerization. Emerging evidence also highlights the contribution of lipid raft‐associated membrane domains, enriched in sphingomyelin, ceramide, cholesterol, and caveolin‐1 (CAV1), which provide structurally ordered regions that facilitate membrane curvature, cargo clustering, and possibly serve as assembly sites for ESCRT components. These raft microdomains are thought to underlie the common lipid composition of EVs, suggesting that EVs may partially originate from raft‐associated regions of the endosomal or plasma membrane [7, 8]. A thorough understanding of these natural sorting mechanisms is fundamental to unraveling the biological roles of EVs. This chapter is organized by cargo type and provides a systematic elucidation of the sorting pathways for proteins, nucleic acids, including miRNA and mRNA, during the process of EV generation.

### 2.1 Mechanisms of Protein Sorting into EVs

The sorting of proteins into ILVs is a highly selective process driven primarily by the ESCRT machinery. This canonical pathway is initiated by the recognition of ubiquitinated transmembrane proteins, which are tagged by E3 ubiquitin ligases at the plasma membrane or early endosomal surface [8]. ESCRT‐0 (HRS and STAM) recognizes these ubiquitin tags, while ESCRT‐I (including TSG101) and ESCRT‐II provide the structural scaffold required for membrane curvature and cargo concentration [9]. Notably, HSP90 can interact with TSG101 to enhance its stability, thereby promoting EV formation under specific signaling conditions. The process culminates as ESCRT‐III (CHMP proteins) polymerizes into helical filaments at the vesicle neck to mediate membrane scission, followed by the AAA ATPase VPS4 disassembling the complex to recycle its subunits.

In addition to the canonical ESCRT machinery, mammalian cells utilize the Syndecan‐Syntenin‐ALIX axis as a major alternative route. In this pathway, Syntenin links the cytoplasmic tail of syndecan‐1 to ALIX, which then bridges the cargo to the ESCRT‐III subunit CHMP4B and the lipid lysobisphosphatidic acid (LBPA). This pathway mediates the selective inclusion of proteins like CD63, β‐integrins, PD‐L1, and viral components such as EBV LMP1, and is regulated by SRC kinase‐mediated phosphorylation and PLD2‐generated phosphatidic acid, both of which promote ALIX recruitment and membrane curvature [10]. Furthermore, sorting can proceed through lipid‐driven and tetraspanin‐mediated mechanisms. Neutral sphingomyelinase 2 (nSMase2) converts sphingomyelin into ceramide, a conical lipid that spontaneously induces negative membrane curvature to promote inward budding [11]. Simultaneously, tetraspanins (CD9, CD63, CD81) organize the membrane into Tetraspanin‐Enriched Microdomains (TEMs), acting as scaffolds to cluster partner proteins. These proteins can interact with partners such as EWI‐2, PTGFRN, and integrins to cluster cargos and regulate membrane organization. For example, CD63 has been reported to promote the incorporation of apolipoprotein E and lysyl‐tRNA synthetase into ILVs. In addition, some cytosolic proteins containing KFERQ motifs can be selectively packaged into EVs through an ESCRT‐independent pathway involving LAMP2A, HSC70, CD63, Alix, Syntenin‐1, Rab31, and ceramides. This pathway enables the transfer of functional proteins such as HIF1A [12]. Such lipid‐ and protein‐based mechanisms thus complement the ESCRT system, ensuring efficient and context‐dependent ILV formation and selective cargo packaging.

### 2.2 Mechanisms of Selective RNA Loading and RBP Coupling

EVs contain a wide range of RNA species, including small non‐coding RNAs (sncRNAs), transfer RNAs (tRNAs), ribosomal RNAs (rRNAs), messenger RNAs (mRNAs), and long non‐coding RNAs (lncRNAs). Among them, sncRNAs are particularly enriched, with miRNAs being the most abundant and well‐studied EV RNA cargos. Current evidence suggests that EV RNA loading occurs through both passive and selective processes. Some RNAs are incorporated randomly according to their abundance in the cytoplasm of parental cells. In contrast, other RNA species are actively enriched through regulated sorting pathways. As a result, the RNA composition of EVs does not simply mirror the cellular transcriptome but instead represents a selectively organized subset of cellular RNAs. This selective loading process is controlled by interactions between RNAs and specific molecular machinery that recognizes sequence or structural features of the cargos.

The enrichment of miRNA in EVs is largely regulated by RNA‐binding proteins (RBPs) involved in both miRNA processing and transport. Argonaute 2 (AGO2), a core component of the RNA‐induced silencing complex (RISC), has been shown to mediate the loading of miRNAs such as let‐7b and miR‐100 into EVs, often together with AGO2‐containing RNP complexes [13, 14]. Neutral sphingomyelinase 2 (nSMase2) also contributes to miRNA sorting through ceramide‐dependent pathways [15]. In addition, membrane‐associated proteins such as CAV1 can cooperate with RBPs, including hnRNPA2B1 and hnRNPA1, to promote selective miRNA packaging [16]. Other machinery components, such as VPS4A, further refine this selection by ensuring the spatial coupling of RNP complexes with the ESCRT‐driven membrane remodeling process. The selectivity of miRNA sorting is further dictated by specific nucleotide sequences or structural motifs that serve as molecular “zip codes” for export. Recent studies have revealed that a significant portion of miRNAs exhibit polarized distribution, being either selectively exported into EVs or actively retained within the cytoplasm. This regulation is mediated by conserved short nucleotide motifs, typically 4–6 nucleotides in length, located at the 3’ end or within loop structures. Specifically, EXOmotifs (such as CGGGAG) promote exosomal export by enhancing RBP‐miRNA interactions, whereas CELLmotifs (such as AGAAC, CAGU, or AUUA) facilitate cellular retention [17]. Moreover, the functionality of these motifs is dependent on their interaction with specific cellular RBPs, such as hnRNPA2B1 [16] and SYNCRIP [18].

Similar to small RNAs, mRNA loading into EVs is also tightly regulated by RBPs that recognize defined sequences or structural elements. Many of these signals are located within the 3’ untranslated region (3’UTR). For example, a 25‐nucleotide “zip‐code” sequence containing a CTGCC core motif can form stem‐loop structures that facilitate mRNA sorting in certain cellular contexts [19]. YBX1 is considered an important regulator of this process and participates in the transport of multiple long RNA species, including mRNAs involved in tissue repair and cell signaling, into exosomal pathways [20]. Proteins such as Annexin A2 and VAMP3 may further support the association of mRNA‐RNP complexes with endosomal or plasma membranes. Moreover, mRNA loading can involve distinct structural interactions, such as high‐affinity binding to ALIX via specific motifs, or the utilization of retroviral‐like mechanisms where proteins like Gag recognize encapsulation signals to direct long RNAs toward budding vesicles. Collectively, these pathways demonstrate that mRNA sorting into EVs is regulated through multiple coordinated, protein‐dependent pathways rather than through a single mechanism [21].

## 3 Engineering Strategies of EVs for Cargo Loading

EVs can be engineered through two principal strategies, namely exogenous modification and endogenous cell bioengineering. Exogenous modification involves the physical or chemical manipulation of purified EVs after isolation. Common methods include co‐incubation (passive diffusion of small hydrophobic drugs into the lipid bilayer), electroporation (transient pore formation by electric pulses to facilitate cargo entry), sonication (ultrasound‐induced deformation of the EV membrane to enhance cargo permeability), freeze–thaw cycling (repeated rapid freezing and thawing to transiently disrupt membrane integrity for cargo entry), and surface conjugation (noncovalent or covalent attachment of functional molecules to the EV membrane surface) [5, 22]. These approaches offer high flexibility and immediacy, enabling direct post‐production customization of vesicle profiles. In contrast, endogenous cell bioengineering harnesses the intrinsic biosynthetic and sorting machinery of parental cells to incorporate therapeutic cargos during EV biogenesis. The choice between exogenous and endogenous strategies is ultimately dictated by the nature of the therapeutic cargo and the intended application. Hydrophobic small molecules are typically loaded into EVs via exogenous co‐incubation, which is more straightforward and less time‐consuming compared to endogenous loading through parental cell processing. Additionally, hydrophilic drugs are usually introduced via physical methods such as sonication, freeze‐thaw cycles, or extrusion, which may compromise membrane integrity or alter membrane structure. Surface conjugation of EVs with dyes, aptamers, or targeting peptides can be achieved through noncovalent (multivalent electrostatic interactions and protein–ligand recognition) or covalent approaches (chemical conjugation, click chemistry, and enzymatic ligation). However, non‐covalent approaches often suffer from reduced stability and specificity in circulation, whereas covalent strategies may be limited by the availability and accessibility of reactive sites on the EV surface, frequently resulting in suboptimal grafting efficiency [23, 24, 25]. For proteins and peptides, endogenous strategies enable their incorporation into both the EV surface and lumen. Surface‐displayed proteins can be utilized for targeting enhancement or receptor‐mediated signaling, whereas encapsulation within the EV lumen is often important for intracellular delivery and functional activity after uptake [26]. In addition, endogenous approaches avoid the introduction of artificial chemical groups and preserve membrane and protein integrity. For nucleic acid cargoes, small species such as siRNA and miRNA can be rapidly loaded exogenously; however, electroporation frequently induces RNA precipitation and aggregation [27]. In contrast, endogenous approaches can protect RNA integrity while achieving higher and more stable loading efficiencies. Long RNA species such as mRNA and circRNA are inherently fragile and susceptible to degradation during exogenous manipulation, making endogenous loading generally the preferred strategy [28]. Similarly, the large size of CRISPR (Clustered Regularly Interspaced Short Palindromic Repeats)‐Cas9 (CRISPR‐associated protein 9) systems poses a considerable barrier to efficient exogenous loading, favoring endogenous encapsulation through scaffold fusions or recruitment tags.

**FIGURE 2:**
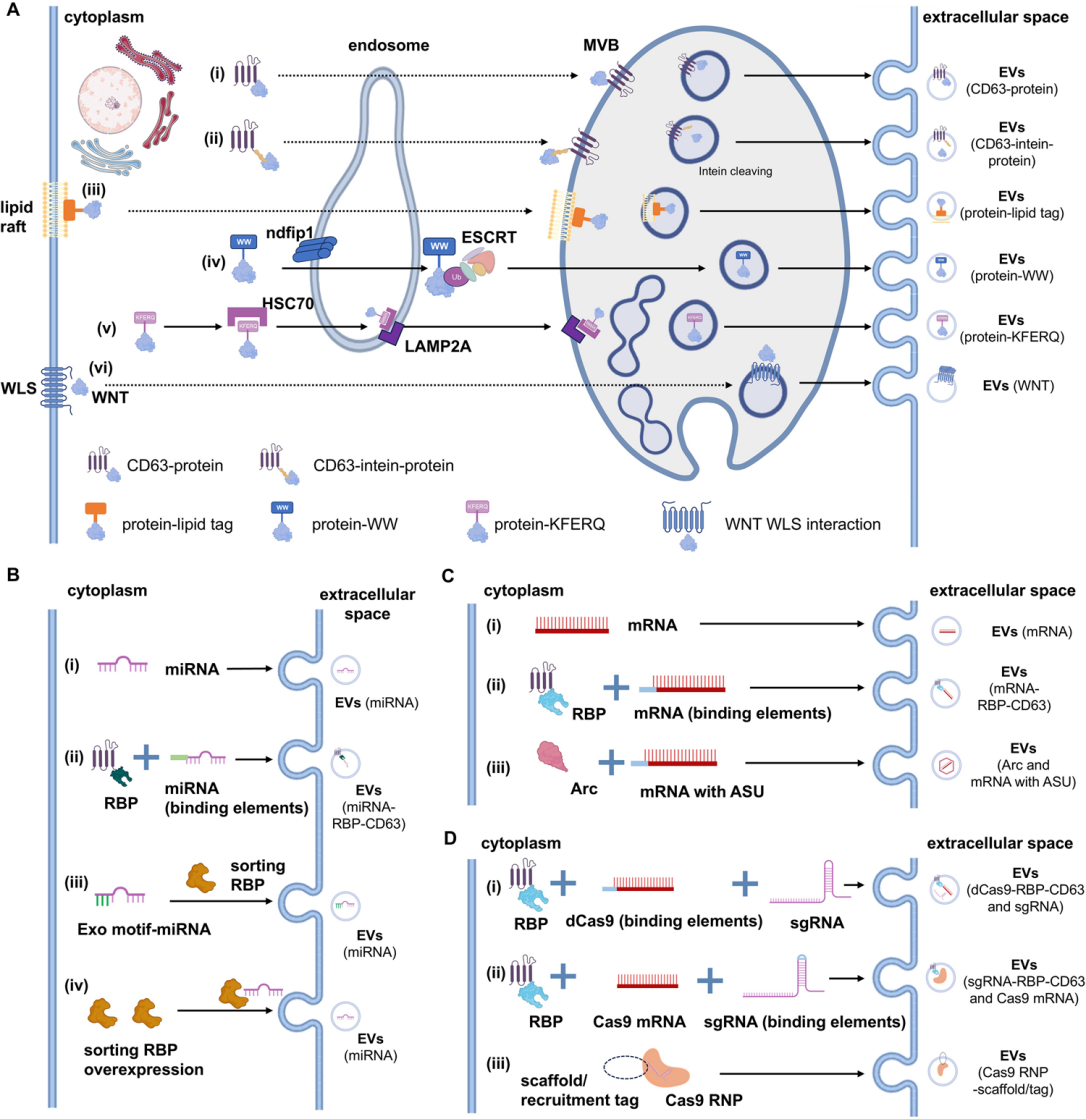
Strategies for engineering EVs to load proteins and nucleic acids, enabled by genetic modulation of EVs‐associated sorting processes. (A) Protein cargo loading strategies. Dashed arrows denote processes not explicitly demonstrated in the referenced studies. (i) Classical scaffold‐mediated loading strategies illustrated using CD63 as an example, in which a protein of interest (POI) is fused to an EV‐enriched scaffold that localizes to MVB membranes and escorts the POI into EVs. (ii) Controlled release strategies, exemplified here by the CD63‐intein‐cargo design, where the pH‐sensitive intein undergoes autocleavage in the acidic MVB environment and detaches the POI from the membrane anchor so that it can be released into the EVs lumen [26]. (iii) Lipidation‐based tag loading strategies in which short lipidation motifs are fused to the POI, leading the lipid‐modified protein to associate with plasma‐membrane lipid rafts and become incorporated into EVs [32]. (iv) WW‐tag‐mediated loading strategies in which a POI fused to a WW motif is recognized by the adaptor Ndfip1, which then recruits Nedd4 family E3 ligases and engages ESCRT machinery to direct the POI into EVs [33]. (v) LAMP2A‐mediated loading strategies in which cargos with KFERQ‐like motifs are recognized by HSC70 and captured by LAMP2A at the endosomal membrane for loading into ILVs [12]. (vi) Fusion‐independent loading strategies exemplified by the WLS‐mediated strategy, in which WLS binds WNT through its natural chaperone function and escorts unmodified WNT into EVs without requiring direct fusion to the POI [34]. (B) miRNA loading strategies. (i) Lentiviral‐mediated delivery of miRNA mimics into parental cells, in which the exogenously introduced miRNAs follow endogenous trafficking pathways and passively enrich in EVs [35]. (ii) Scaffold‐RBPs fusion‐based loading strategies in which an EV‐localized scaffold is fused to RBPs, allowing the RBPs to bind miRNAs containing their cognate recognition sequences and guide them into EVs [36, 37]. (iii) EVs‐enrichment motif‐guided loading strategies in which these motifs are artificially incorporated into target miRNAs, enabling sorting RBPs to recognize them and drive engineered miRNAs packaging into EVs [17]. (iv) RBPs‐overexpression loading strategies in which parental cells overexpress specific sorting RBPs, leading the RBPs to capture their endogenous target miRNAs and promote their accumulation in EVs [16, 38]. (C) mRNA loading strategies. (i) Plasmid or lentiviral‐mediated expression of target mRNAs in parental cells, where the increased intracellular abundance results in passive incorporation of the transcript into EVs [39]. (ii) Scaffold‐RBPs fusion‐based loading strategies in which an EV‐enriched scaffold is fused to an RBP that recognizes defined sequence elements within the target mRNA, thereby directing the RBP‐mRNA complex into EVs [40, 41, 42]. (iii) Arc‐based capsid loading strategies in which mRNAs containing the ASU stabilizing motif are selectively bound by self‐assembling Arc capsid structures and efficiently packaged into engineered EVs [43]. (D) Cas9 RNP loading strategies. (i) RBPs‐guided loading strategies for the CRISPR/dCas9 system, in which a scaffold‐RBP fusion recruits tagged dCas9 mRNA into EVs, enabling intracellular translation and RNP assembly in recipient cells [36]. (ii) RBPs‐guided loading strategies via single‐guide RNA (sgRNA recognition, in which an sgRNA engineered with an RBP‐binding motif associates with the scaffold‐fused RBPs, leading to the loading of the pre‐assembled Cas9 RNP into EVs [44]. (iii) Fusion‐ or tag‐based loading in which Cas9 protein is directly fused to EV‐enriching scaffolds or recruitment tags, exemplified by CD63‐intein‐Cas9, [26] BASP1‐Cas9 fusion [45], and ABI‐tagged Cas9 [46], enabling efficient encapsulation of Cas9 RNPs into EVs.

Without harsh physical or chemical manipulation, endogenous cell bioengineering reduces the risk of EV membrane disruption, better preserves vesicle structural and functional integrity [4, 29], and minimizes cargo damage commonly associated with exogenous loading procedures [30, 31]. Endogenous strategies also possess advantages in terms of translational manufacturing. Environmental stimuli, such as hypoxia or nutrient modulation, can significantly enhance EV yield [25]. Furthermore, by integrating cargo biosynthesis and EV loading into a single continuous bioprocess within stable producer cell lines, endogenous approaches eliminate the multiple steps of post‐isolation cargo loading, thereby improving production efficiency and reproducibility. Given their advantages in preserving vesicle integrity, enabling efficient and stable loading of proteins and nucleic acids, and supporting scalable manufacturing, this section focuses on endogenous cell bioengineering strategies for the incorporation of diverse therapeutic cargos (Figure 2A–D), including proteins, miRNAs, mRNAs, and CRISPR‐Cas9 systems.

### 3.1 Protein Cargo Loading in EVs

Proteins represent one of the most functionally diverse and therapeutically relevant classes of EVs cargos. The native protein trafficking is inherently coordinated with the EV biogenesis, and this physiological synergy makes proteins particularly suitable for endogenous bioengineering. A wide spectrum of engineering approaches has been developed to harness this principle, spanning from global remodeling of the cellular proteome to precise genetic modulation of EV‐associated sorting processes (Table 1). Environmental stimuli can reshape cellular expression programs, resulting in EVs that exhibit corresponding alterations in their protein composition. Besides the utilization of this plasticity, rational genetic engineering allows precise control of protein recruitment through fusion with classical or newly identified scaffold proteins, incorporation of short recruitment tags, or conditional release systems that direct cargos into the vesicle lumen (Figure 2A). More recently, fusion‐independent mechanisms utilizing natural trafficking chaperones or fusogenic envelope proteins have expanded the capacity to load unmodified, functionally intact proteins (Figure 2A). Together, these approaches range from remodeling the parental cells to programming individual molecules, offering flexible strategies to generate EVs with defined compositions and controllable functions.

**TABLE 1:** Protein cargo loading in EVs.

Loading strategy	Specific condition or fusion molecule	Parental cells	Cargo protein	Ref.
Environmental conditioning	Hypoxic condition	Breast cancer cells	GNAI2, ANXA5, TUBB3, TRIP10, CCT7, H2BC12, and LGALS3BP	[47]
	Acidic condition	Melanoma cells	HSP90, NRAS, HRAS, and HYOU1	[48]
	Glutamine‐deficient	Macrophages	MHC class II, CD86, ICAM‐1, and inflammatory mediators	[49]
	Three‐dimensional (3D)‐culture	BM MSCs	Hepatocyte growth factor	[50]
	Silicate ions	Endothelial progenitor cells	VEGF, SDF‐1, CXCR4, and eNOS	[51]
Cargo‐scaffold fusion	CD63‐	HEK293T cells	p53	[52]
	Lamp2b‐	Dendritic cells	RVG peptide	[53]
	C1C2 domain of MFGE8‐	HEK293T cells	EGFR‐binding monobody	[54]
	TSPAN14‐	Expi293F cells	GFP	[55]
	TSPAN2‐, TSPAN3‐	HEK293T cells	ThermoLuc	[56]
	PTGFRN‐, BASP1‐	HEK293 cells	GFP	[45]
	PLXNA1‐	HEK293F cells	GFP	[57]
Cleavable cargo‐scaffold Fusion	CD9‐CIBN, CRY2‐ (EXPLOR)	HEK293T cells	Bax, srIκB, Cre	[58]
	CD9‐CIBN, CRY2‐ (EXPLOR)	HEK293T cells	srIκB	[59]
	CD9‐ mMaple3‐ (MAPLEX)	HEK293T cells	OCT4, SOX2,Cre recombinase	[60]
	CD63‐intein‐	HEK293T cells	Cre recombinase, srIκB	[26]
Recruitment tag‐cargo fusion	Lipidation tags (e.g., palmitoyl‐myristoyl)	HEK293FT cells	synTF	[32]
	Viral late‐domain mimics (WW tag)	LN18 cells	Cre recombinase	[33]
	KFERQ‐like sequences	ARPE‐19 cells	Hypoxia‐inducible transcription factor 1α	[12]
Fusion‐independent loading	WLS‐WNT interaction	HEK293 cells	WNT	[34]

#### 3.1.1 Environmentally Induced Synergistic Remodeling of EV Protein Cargos

Environmental modulation of parental cells profoundly reshapes the protein composition of secreted EVs, offering an indirect route to tailor their biological functions. Environmental cues such as hypoxia, acidosis, nutrient deprivation, culture dimensionality, and chemical stimuli can markedly alter the expression and secretion of functionally relevant proteins, giving rise to EVs with enhanced regenerative, angiogenic, or immunomodulatory properties.

Under hypoxic conditions, breast cancer cells secreted EVs enriched with proteins involved in cell cycle regulation, vesicle‐mediated transport, and Rho GTPase signaling, such as GNAI2, ANXA5, TUBB3, TRIP10, CCT7, H2BC12, and LGALS3BP. These hypoxia‐induced EV proteins may facilitate intercellular communication and enhance the migration of recipient cells [47]. Acidic microenvironments similarly induced melanoma‐derived EVs to accumulate metastatic and pro‐invasive factors, including HSP90, NRAS, HRAS, and HYOU1, thereby enhancing migration and angiogenic signaling in recipient cells [48]. Nutrient deprivation profoundly influences EV composition, as macrophages under glutamine‐deficient conditions released EVs enriched in MHC class II, CD86, ICAM‐1, and inflammatory mediators that collectively enhanced cytokine secretion and T‐cell activation [49]. Structural and chemical factors also shape the protein content of EVs. For example, three‐dimensional (3D)‐cultured bone marrow‐derived mesenchymal stem cells (MSCs) produced EVs enriched in hepatocyte growth factor, promoting cytoprotection and proliferation [50], whereas exposure of endothelial progenitor cells (EPCs) to silicate ions enhanced vesicle secretion and incorporated angiogenic mediators such as VEGF, SDF‐1, CXCR4, and eNOS, thereby facilitating neovascularization and cardiac repair in vivo [51]. It is important to note that the specific sorting routes for these individual proteins were not directly traced in the original studies. Instead, the observed enrichment was identified retrospectively through comparative proteomic analyses of EVs derived from stimulated versus control cells, with subsequent functional validation of the differentially abundant proteins. Therefore, these enriched proteins are presumed to enter EVs via the canonical trafficking pathways through the endosomal‐MVB route. Consistent with this notion, their sub‐vesicular localization mirrors that of their native forms: cytosolic proteins such as HSP90 reside within the EV lumen, whereas transmembrane proteins like MHC class II and CD86 are displayed on the EV surface.

This coordinated proteomic remodeling is not limited to functional cargo proteins alone; the same environmental cues also modulate the expression of key components involved in EV biogenesis and secretion. Hypoxia elevates tetraspanins (CD63, CD81) and Rab27a/b while modulating ESCRT components (TSG101 and ALIX) in a context‐dependent manner [48, 61, 62]. Nutrient deprivation, conversely, transcriptionally upregulates ESCRT‐related genes and promotes vesicle budding efficiency [49]. Stress conditions also enhance the incorporation of chaperones such as HSP70, HSP90, and Annexin II, reflecting cytoskeletal remodeling during vesicle formation [48, 61, 62]. Such environmentally induced changes in EV biogenesis pathways may act synergistically with scaffold‐based loading strategies to improve both the yield and functional performance of engineered EVs.

#### 3.1.2 Scaffold‐Mediated Fusion Strategies for Selected Protein Loading

Beyond environment‐induced remodeling, more targeted engineering strategies have been established to endow EVs with selected therapeutic cargos. When a specific protein of interest (POI) is identified, genetic fusion to EV‐enriched scaffold proteins provides a controllable means of achieving selective and reproducible incorporation. Through this approach, the POI is physically linked to endogenous membrane or endosomal components, allowing it to be actively recruited into budding vesicles via native sorting pathways rather than being passively included during secretion.

The endogenous components most commonly exploited for this purpose, collectively referred to as scaffolds, serve as molecular vehicles to escort fused cargoes into vesicles. Classical scaffolds can be broadly categorized into three classes (Figure 2A), namely tetraspanins, type I transmembrane proteins, and peripheral membrane proteins. Tetraspanins, exemplified by CD63, CD9, and CD81, are incorporated into EVs through two major mechanisms. They can recruit ALIX and engage the ESCRT‑III machinery for sorting into ILVs within MVBs [8], as well as self‐associate into tetraspanin‐enriched microdomains that facilitate ESCRT‐independent vesicle budding [63, 64, 65]. In EVs, tetraspanins adopt a topology with both N‑ and C‑termini facing the intravesicular lumen and two extracellular loops displayed on the surface. As a result, fusion at either terminus localizes cargos to the lumen, whereas insertion into the second extracellular loop enables surface display on EVs. A representative application is the fusion of the tumor suppressor p53 to the C‐terminal of CD63, which facilitated its efficient incorporation into EV lumen and functional delivery to cancer cells, ultimately inducing apoptosis [52]. Type I transmembrane proteins, such as Lamp2b and PDGFR, are sorted into EVs through distinct mechanisms involving either cytoplasmic sorting motifs or transmembrane domain‐mediated recognition [45, 53, 66]. Lamp2b is directed into ILVs via a conserved tyrosine‑based sorting motif (YXXΦ) in its C‑terminal tail, recognized by adaptor protein complexes on MVB membranes [67]. In contrast, PDGFR mainly relies on its transmembrane domain for EV sorting, and this domain alone is sufficient to achieve efficient EV enrichment [68]. As single‑pass transmembrane proteins, these scaffolds insert into the EV membrane with their N‑termini exposed on the exterior and C‑termini in the lumen, meaning that cargos fused to the N‑terminus are presented on the EV surface. For instance, conjugation of the rabies viral glycoprotein (RVG) peptide to Lamp2b has been shown to endow EVs with neuron‑specific tropism, thereby enabling targeted delivery to the central nervous system [53]. Peripheral membrane proteins, such as MFGE8 and Syntenin, attach to EV membranes through lipid‑binding or adaptor linkages [69]. The C1C2 domain of MFGE8 directly recognizes phosphatidylserine on the EV membrane, enabling stable membrane association [70]. Syntenin binds the cytoplasmic domain of transmembrane syndecan and subsequently recruits ALIX, thereby coupling the complex to the ESCRT machinery for sorting into ILVs [10]. Because peripheral scaffolds are not embedded in the lipid bilayer, cargos fused to them are typically localized to the membrane‑associated surface of the EV. One representative example is the fusion of an EGFR‐binding monobody to the C1C2 domain of MFGE8, which enabled the surface presentation of targeting ligands and improved tumor‐selective binding [54]. Collectively, these examples establish scaffold‑based fusion as a cornerstone of endogenous protein loading. Importantly, the final sub‐EV localization of cargos can be tuned by both the intrinsic sorting properties of the scaffold and the topology determined by fusion design.

Systematic quantitative studies have subsequently characterized the loading behavior of classical scaffold proteins, providing a foundation for rational optimization. Using GFP as a model cargo, Corso et al. quantified GFP‐positive (GFP^+^) EVs by fluorescence‐based nanoparticle tracking analysis (NTA) and found that fusion of GFP to the N‐terminus of tetraspanins CD63, CD9, and CD81 yielded GFP^+^ EV fractions of approximately 25.8%, 29.5%, and 9.5%, respectively [69]. In contrast, the membrane‐associated MFGE8 produced nearly 0%, while soluble proteins such as syntenin achieved 15.4% positivity. Single‐vesicle nanoflow cytometry further confirmed this heterogeneity. Silva et al. reported that modified constructs such as CD63TM and CD81TM (C‐terminally fused with the PDGFRβ transmembrane domain) generated roughly 30% GFP^+^ EVs [55], whereas Dooley et al. showed that CD63, CD9, and CD81 achieved moderate efficiencies of 51.5%, 63.7%, and 57.1%, respectively, compared with ∼10.5% for Lamp2b [45]. Protein topology was another key determinant of loading efficiency. Corso et al. observed that GFP fusion at either the N‐ or C‐terminus of CD63 generated around 25% GFP^+^ EVs, while insertion into the second loop reduced this to around 2% [69]. Conversely, Silva et al. found that placing GFP within the second loop of CD63 yielded approximately 35% GFP^+^ EVs, surpassing the CD63TM construct [55]. In addition to population‐level differences, some of the above studies also quantified the average number of cargo molecules per vesicle. By combining NTA for particle counts with GFP ELISA, CD63‐, CD9‐, and CD81‐based constructs were estimated to carry about 50–100 GFP molecules per EV [45]. Single‐molecule localization microscopy further refined these measurements, revealing mean loadings of 103 for CD81TM, 70 for CD63, and 50 for CD63TM [55]. Collectively, these findings establish quantitative single‐vesicle benchmarks for evaluating scaffold‐dependent loading efficiency. Because these values were obtained using diverse analytical platforms and experimental conditions, direct numerical comparisons across studies should be made with caution. Nevertheless, consistent intra‐study trends (such as the relative performance of scaffold proteins measured under identical conditions) remain informative and serve as practical references for optimizing scaffold selection and improving EV‐loading strategies.

The above systematic analyses indicate that classical scaffold systems produce heterogeneous EV populations with varying cargo content. To enrich functional vesicles and improve loading efficiency, tagging‐based strategies have been explored. Obuchi et al., for example, engineered CD63 with a surface 3×FLAG tag, allowing magnetic immunocapture of FLAG^+^ EVs and enrichment of functional mCherry^+^ vesicles to approximately 90% [71]. In parallel, researchers have explored novel scaffolds with higher loading capacity and improved uniformity. Silva et al. developed a quantitative workflow combining bioinformatic screening and single‐vesicle imaging, identifying TSPAN14 (a tetraspanin localized to the EV membrane) as an efficient tetraspanin capable of intraluminal loading of approximately 173 GFP molecules per EV, substantially exceeding CD63 or CD81 [55]. Similarly, Zheng et al. screened 244 EV‐associated candidates using a ThermoLuc‐based assay and found that the tetraspanins TSPAN2 and TSPAN3 produced 1.6‐ and 1.3‐fold higher per‐particle luminescence than CD63‐based constructs [56]. These newly identified tetraspanins displayed distinct surface proteomic signatures and appeared to function independently of the classical CD9/CD63/CD81 network, thereby reducing potential competition for shared sorting pathways. Expanding beyond the tetraspanin family, Dooley et al. used LC‐MS/MS proteomics to identify PTGFRN (a type I transmembrane IgSF‐EWI protein) and BASP1 (a lipid‐anchored MARCKS‐family protein) as highly efficient scaffolds [45]. As a type I transmembrane protein, PTGFRN localizes cargo to the EV surface via N‐terminal fusion. BASP1, in contrast, relies on its N‑terminal myristoylation and polybasic region to stick to the inner leaflet of the EV membrane. By fusing cargo to the C‑terminus of the BASP1 fragment, the cargo gets packed into the EV lumen. EVs engineered with these proteins exhibited over 95% cargo‐positive vesicles, carrying roughly 400 and 1,000 cargo molecules per EV, respectively, which represented a substantial increase compared with traditional scaffolds (such as CD9, CD63, CD81, and Lamp2b). Zhao et al. refined the selection framework by requiring candidates to be type I transmembrane proteins with intrinsic EV‐sorting activity and to retain this activity after N‐ or C‐terminal truncation or fusion [57]. Through this framework, a truncated variant of PlexinA1 (PLXNA1(863‐1316)) was identified as an exceptionally potent scaffold protein, achieving more than a threefold increase in mean fluorescence intensity over PTGFRN‐EVs in HEK293 cells. This efficiency was attributed to a conserved SDSLLTL motif adjacent to the transmembrane region, deletion of which abolished EV enrichment. Although the precise downstream sorting mechanism remains unclear, this motif is required for the efficient incorporation of PLXNA1 fusion proteins into EVs. Fusion of a POI to the N‐terminus enables its display on the EV surface, as demonstrated for IL‐12 and nanobodies, whereas fusion to the C‐terminus localizes the POI within the EV lumen, as shown for the RNA‐binding protein L7AE.

Moreover, recent engineering breakthroughs have expanded the scaffold paradigm beyond endogenous host proteins to include potent viral fusogenic proteins. The vesicular stomatitis virus glycoprotein (VSV‐G), a trimeric class III viral envelope protein renowned for mediating robust endosomal escape through low‐pH‐triggered membrane fusion [72], has been repurposed as a highly efficient, multifunctional EV‐sorting scaffold. In a pivotal study by Liang et al., VSV‐G was demonstrated to function as an EV‐sorting domain, analogous to classical scaffolds like CD63 [26].

Taken together, these advances demonstrate that newly identified scaffolds markedly enhance cargo loading efficiency and population uniformity. Future work may combine high‐throughput proteomic analyses with rational selection criteria to pinpoint proteins with intrinsic EV‐sorting ability and structural compatibility for fusion. These candidates should then be validated through quantitative single‐vesicle assays to define their loading capacity, topology, and functional integrity.

#### 3.1.3 Controlled Release of Protein Cargos Into the EV Lumen

The scaffold‐mediated fusion strategies lack an efficient mechanism to separate cargo proteins from EV membrane anchors, which limits cytosolic delivery efficiency [58]. Recent studies have explored controlled‐release strategies that actively promote cargo release into the EV lumen under specific environmental conditions. These approaches are designed to improve both cargo loading and protein activity by releasing cargos from scaffold anchors during EV biogenesis, allowing soluble proteins to accumulate within the EV lumen before uptake by recipient cells.

**FIGURE 3:**
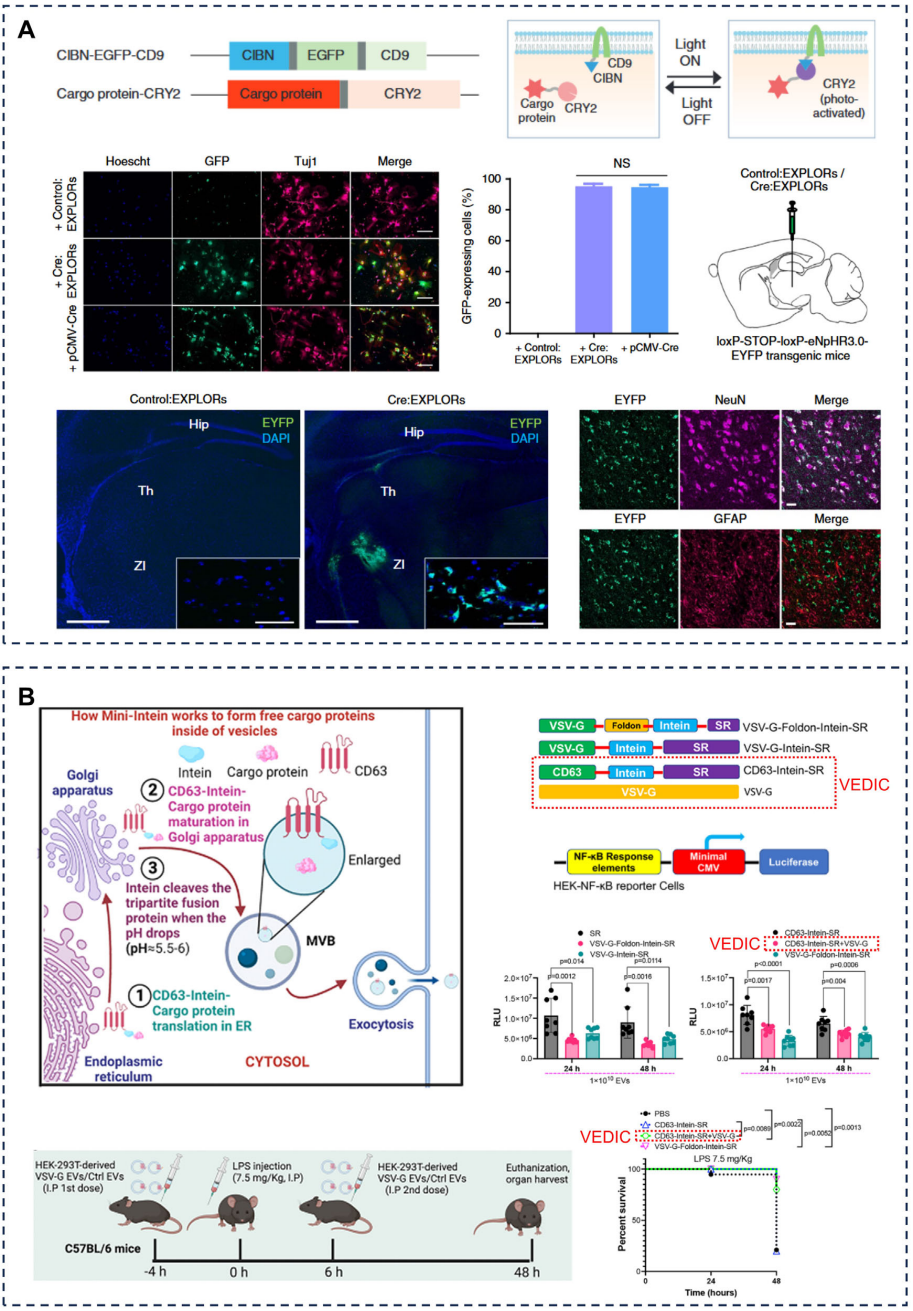
Controlled release of protein cargos into the EV lumen. (A) Schematic illustration of the design of the EXPLOR system and its application for the functional intracellular delivery of proteins, as demonstrated by EXPLOR‐mediated delivery of Cre recombinase in vitro and in vivo. Reproduced with permission [58]. Copyright 2016, Springer Nature. B) Schematic illustration of the design and application of the VEDIC system, demonstrating soluble cargo loading via a pH‐sensitive intein and its application in therapeutic protein delivery. The platform was used to load a super‐repressor of NF‐κB, which was functionally validated through inhibition of NF‐κB signaling in reporter cells and significantly improved survival in a murine model of LPS‐induced systemic inflammation. Reproduced with permission [26]. Copyright 2025, Springer Nature.

The EXPLOR (exosomes for protein loading via optically reversible protein–protein interactions) system represents an innovative optogenetically engineered platform for efficient intracellular protein delivery. This system is built by engineering HEK293T cells to co‐express two key fusion proteins: a cargo protein fused to the photoreceptor CRY2 (cryptochrome 2), and the classical scaffold protein CD9 fused to CIBN, a truncated version of CIB1 (cryptochrome‐interacting basic helix‐loop‐helix 1). Under blue light illumination, CRY2 undergoes a conformational change and binds to CIBN, thereby recruiting the cargo protein to the endosomal membranes where EVs are formed. When light exposure is terminated, the CRY2‐CIBN interaction dissociates, releasing the cargo into the lumen of newly formed EVs. This platform enabled the delivery of diverse functional proteins, from pro‐apoptotic Bax and super‐repressor IκB (srIκB) for modulating cell death and signaling pathways, to Cre recombinase for efficient genome editing. Cre‐loaded EXPLOR (Cre:EXPLOR) achieved recombination in over 95% of cultured primary neurons, which are notoriously difficult to transfect. Furthermore, upon stereotactic injection into the mouse brain, Cre:EXPLORs successfully delivered functional Cre protein, leading to robust transgene expression in neurons (Figure 3A) [58]. Subsequently, the EXPLOR system was used to deliver srIκB to septic mice, significantly improving survival and alleviating organ damage by suppressing the inflammatory response [59]. Besides, Han et al. designed a controllably releasable system termed MAPLEX (mMaple3‐mediated protein loading into and release from exosomes), which requires only a single light activation step before EVs administration [60]. In this design, the photocleavable fluorescent protein mMaple3 (a Clavularia‐derived green‐to‐red photoconvertible variant) is inserted as a switch between the cargo protein and CD9. MAPLEXs are produced without light, and the cargo is efficiently released into the intravesicular space by a single, brief 405‐nm light illumination after EVs isolation. This approach has been successfully applied to deliver a range of functional proteins, including transcription factors (OCT4, SOX2) and Cre recombinase, demonstrating its versatility for intracellular protein delivery.

In addition to external stimuli, endogenous physicochemical cues have been harnessed to enable autonomous protein release. Liang et al. utilized an engineered mini‐intein from Mycobacterium tuberculosis recA that undergoes pH‐sensitive C‐terminal cleavage at 37°C (Figure 3B) [26]. They designed a fusion construct following the CD63‐intein‐cargo architecture, wherein therapeutic proteins such as Cre recombinase and srIκBα were linked to the EV‐sorting protein CD63. During EV biogenesis, the acidic environment of MVB promotes protonation of key residues in the intein active site, triggering autocleavage and resulting in the encapsulation of soluble, functionally active proteins within the EV lumen. To further improve cytosolic delivery after cellular uptake, the system additionally incorporated the fusogenic protein VSV‐G. This engineered platform, termed VEDIC (VSV‐G plus EV‐Sorting Domain‐Intein‐Cargo), enhances endosomal escape and promotes cytosolic release of cargos. Consequently, EVs loaded with Cre recombinase mediated highly efficient gene recombination, while srIκBα delivery via engineered EVs conferred significant protection and improved survival in a murine model of LPS‐induced systemic inflammation.

In these controlled‐release systems, cargo proteins are initially directed into the EV biogenesis pathway through fusion with tetraspanin scaffolds such as CD9 or CD63. Because the N‐ and C‐termini of tetraspanins face the cytosol, and subsequently the EV lumen after vesicle formation, cargos are typically fused to these intraluminal regions. The incorporation of cleavable or reversible linkers allows cargos to dissociate from membrane anchors and accumulate as soluble proteins within the EV lumen. Such strategies not only promote efficient uptake and release of EV contents into recipient cells but also avoid intracellular entrapment caused by protein anchoring. This provides a robust and versatile strategy for the delivery of therapeutic proteins, especially those requiring cytosolic or nuclear localization to function.

#### 3.1.4 Recruitment Tags‐Guided Protein Loading

In addition to fusing cargos to EV scaffold proteins, another strategy uses short peptide tags that naturally interact with the vesicle biogenesis machinery. These tags act as molecular targeting signals that connect POIs with endogenous trafficking pathways, thereby promoting their incorporation into EVs without the need for fusion to large membrane scaffolds.

**FIGURE 4:**
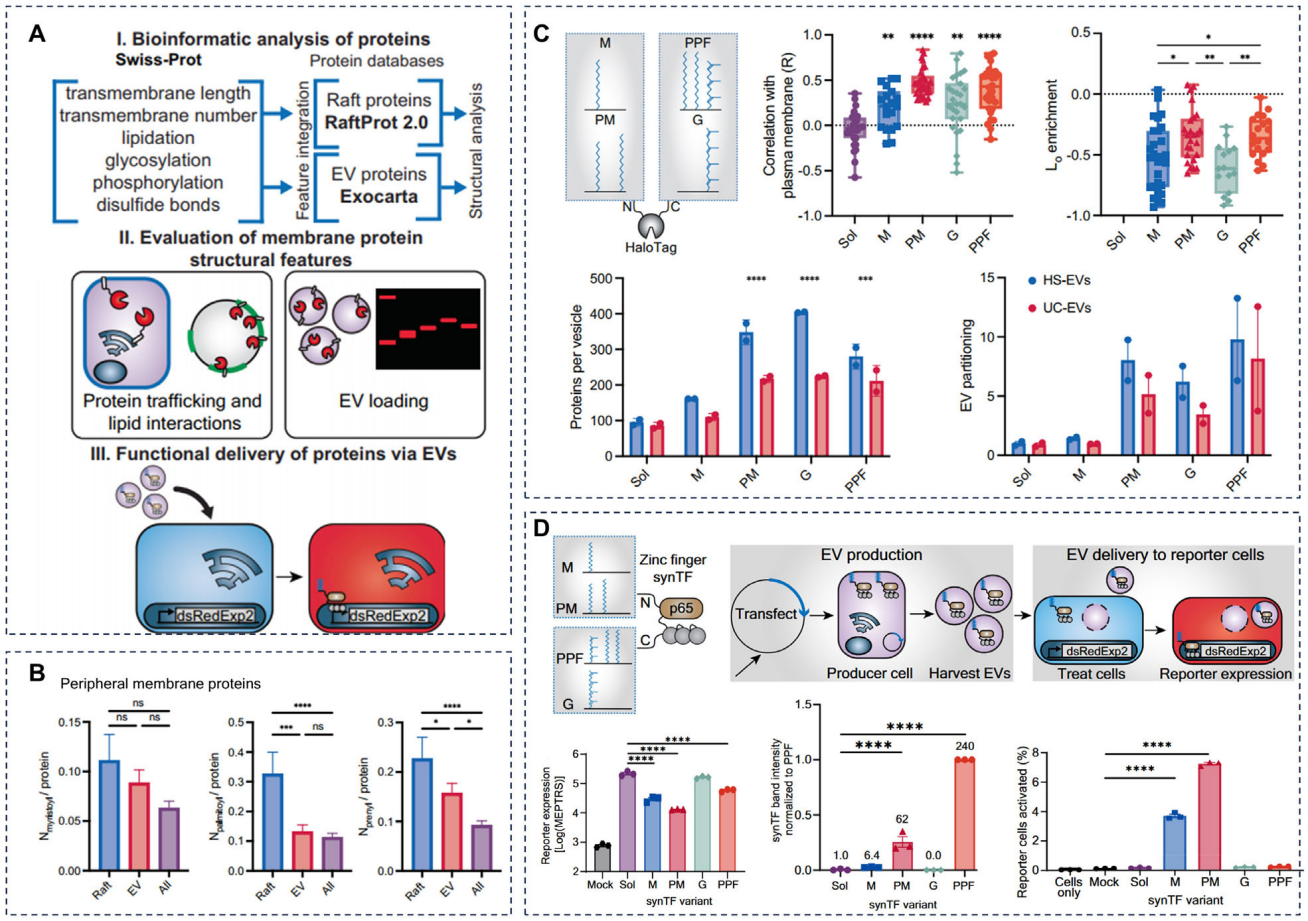
Rational engineering of lipidation‐based tags for protein cargo loading and functional delivery. Reproduced with permission [32]. Copyright 2024, Springer Nature. (A) Overview of the rational engineering workflow, from bioinformatic analysis to functional validation. (B) Bioinformatic evidence that peripheral membrane proteins naturally enriched in EVs frequently bear myristoylation, palmitoylation, or prenylation modifications. (C) Experimental demonstration that palmitoylation‐containing tags (PM, PPF) enhance plasma membrane localization, lipid raft association, and EV loading efficiency using HaloTag as a model cargo. (D) Validation with a synthetic transcription factor (synTF), showing that the PM tag achieves the optimal balance between efficient EV encapsulation and functional gene activation in recipient cells.

Lipidation‐based tags are one representative example. Peruzzi et al. demonstrated that EV membranes share close structural similarity with plasma‐membrane lipid rafts in terms of membrane order and protein composition [32]. By cross‐referencing the RaftProt 2.0, ExoCarta, and Swiss‐Prot databases, the authors found that peripheral membrane proteins enriched in EVs frequently contain lipid modifications such as myristoylation, palmitoylation, and prenylation (Figure 4A,B). Building on this observation, the authors genetically fused short peptide sequences encoding endogenous lipidation signals, termed lipidation tags, to selected proteins such as HaloTag. These tags, including myristoyl (M), palmitoyl‐myristoyl (PM), geranylgeranyl (G), and palmitoyl‐palmitoyl‐farnesyl (PPF), serve as recognition sites for host enzymes that attach fatty acyl or prenyl groups, thereby anchoring the proteins to raft‐like membrane domains and facilitating their enrichment in EV membranes (Figure 4C). Importantly, because these lipidation reactions occur on the cytosolic face of cellular membranes, the modified protein becomes anchored to the inner leaflet of the plasma membrane. During EV biogenesis, these membrane‐associated cargos are carried into budding vesicles and ultimately localize to the luminal side of the EV membrane. Using a synthetic transcription factor (synTF) as a model cargo, the authors first confirmed that while these lipid tags variably impaired its intrinsic transcriptional activity upon direct expression in cells, all variants retained substantial functionality (Figure 4D). The strength of this membrane association determines the loading efficiency. Tags with higher affinity, particularly PM and PPF, produced greater EV incorporation of fused cargos. However, only the PM‐tagged synTF activated reporter gene expression in recipient cells, whereas the more tightly bound PPF variant produced minimal functional delivery, likely because its strong anchoring hindered cytosolic release after membrane fusion. Collectively, this work provides a general strategy for loading soluble or peripheral proteins into EVs. Different lipidation tags confer different membrane affinities, which directly affect both EV loading efficiency and cargo release after delivery. Stronger membrane association generally improves EV incorporation, but may hinder intracellular release and reduce biological activity. Therefore, effective EV engineering requires a balance between efficient loading and efficient cargo release after uptake. This concept is not limited to lipidation tags and may also guide the design of other EV‐based protein delivery systems.

Other tags are derived from viral budding mechanisms that hijack the ESCRT machinery. During both viral budding and endosomal vesicle formation, conserved late‐domain (L‐domain) motifs serve as docking elements for ESCRT adaptors. Specifically, PPxY motifs interact with WW‐domain‐containing E3 ubiquitin ligases of the Nedd4 family [73]; PT/SAP binds directly to TSG101 (a component of ESCRT‐I) [74]; and YPX(n)L engages ALIX [75], an ESCRT‐associated protein. Through these interactions, membrane‐associated cargos can be linked to the ESCRT machinery and sorted into ILVs [76]. Building upon this concept, Sterzenbach et al. engineered soluble proteins by fusing a minimal WW tag, which is recognized by the adaptor Ndfip1 carrying PPxY motifs [33]. Ndfip1 subsequently recruited Nedd4 ubiquitin ligases, triggering ESCRT engagement and vesicle budding, which led to efficient packaging of soluble cargos such as Cre recombinase into secreted EVs and enabled their functional delivery both in vitro and in vivo. Notably, protease protection assays confirmed that the WW‑tagged Cre protein is localized inside the EVs lumen, as it was resistant to proteinase K digestion unless the vesicle membrane was permeabilized with Triton X‑100.

In addition, Ferreira et al. identified a chaperone‐mediated loading mechanism that operated independently of ESCRT at early endosomes [12]. Proteins containing KFERQ‐like sequences were recognized by HSC70 and captured by Lamp2a, which, together with CD63, ALIX, Syntenin‐1, and Rab31, directed their sorting into ILVs. Artificial addition of these KFERQ‐like tags to cytosolic proteins markedly enhanced their encapsulation into EVs and enabled efficient transfer of functional cargos such as hypoxia‐inducible transcription factor 1α, both in vitro and in vivo. Trypsin protection assays further confirmed that these cargos were enclosed within the EV lumen.

Collectively, these studies illustrate how recruitment tag‐based strategies enable flexible control over protein localization and release within EVs. In the systems developed by Sterzenbach et al. and Ferreira et al., the appended peptide tags mediate the selective recruitment of soluble proteins into the intraluminal space of EVs, where cargos remain freely diffusible and can be readily released into recipient cells to exert their activity [12, 33]. In contrast, the lipidation tag strategy described by Peruzzi et al. anchors the cargo to the EV membrane through covalent lipid modifications [32]. Although this membrane association restricts complete release, the use of tags with graded membrane affinities offers a tunable balance between efficient loading and functional delivery. Together, these studies show that different recruitment tags can direct cargos into either a freely soluble luminal state or a membrane‐associated state within EVs, which subsequently affects cargo release in recipient cells.

#### 3.1.5 Fusion‐Independent Strategies for Protein Loading

While scaffold‐ or tag‐based strategies significantly enhance the efficiency of EV cargo loading, the required covalent fusion of the POI to a scaffold or tag may unintentionally alter its structural integrity or biological function. Such modifications may disrupt native folding, interfere with post‐translational modifications, or block important trafficking and interaction domains. For instance, tagged WNT proteins have been shown to display markedly reduced signaling activity compared with their native counterparts [77]. Yang et al. thus developed a fusion‐independent strategy that harnesses a specific physiological chaperone‐cargo interaction [34]. Rather than relying on generic engineered scaffolds or tags, this method exploited Wntless (WLS), an eight‐pass transmembrane chaperone that naturally escorts lipid‐modified WNT ligands from the Golgi to the plasma membrane. By co‐expressing WLS and WNT proteins in parental cells, WLS was repurposed as an intrinsic EV‐sorting receptor, enabling efficient, fusion‐free loading of WNT ligands into EVs. Immunogold labeling further confirmed that the loaded WNT3A molecules are displayed on the EVs surface rather than encapsulated in the lumen. The resulting WLS‐engineered EVs effectively incorporated WNT3A and activated WNT/β‐catenin signaling to a level comparable to recombinant WNT3A, indicating that protein structure and biological activity were largely preserved. This strategy expands current EV loading approaches by utilizing endogenous chaperone‐cargo interactions instead of artificial fusion design, thereby helping maintain native protein functionality. However, the WLS‐based system is still highly cargo‐dependent and may require identification of additional chaperone‐cargo pairs for broader application to other therapeutic proteins.

### 3.2 Nucleic Acid Cargo Loading in EVs

EVs have emerged as versatile carriers for nucleic acid‐based therapeutics, offering protection from enzymatic degradation and enabling biologically active transfer to target cells. Given the vast diversity of nucleic acid size, complexity, and intracellular behavior, EV‐based nucleic acid loading strategies can be broadly categorized into three classes (Table 2): (1) small RNAs such as miRNAs, whose intracellular abundance and sorting can be modulated by environmental stimuli or genetic engineering (Figure 2B); (2) mRNAs, which require more elaborate recruitment mechanisms, often mediated by RBPs or virus‐inspired modules (Figure 2C); and (3) RNP complexes, such as the Cas9 RNP (the core of the CRISPR‐Cas9 system), whose multi‐component assembly necessitates synthetic scaffolds or inducible tethering architectures (Figure 2D). Regardless of the specific loading strategy, all nucleic acid cargos discussed in this section are ultimately encapsulated within the EV lumen, where the lipid bilayer provides protection from extracellular nucleases.

**TABLE 2:** Nucleic acid cargo loading in EVs.

Loading strategy	Specific condition or fusion molecule	Parental cells	Cargo nucleic acid	Ref.
Environmental conditioning	Hypoxic preconditioning	Cardiac progenitor cells	miR‐15b, miR‐17, miR‐20a, miR‐103, miR‐199a, miR‐210, and miR‐292	[78]
Environmental conditioning	3D culture	MSCs	miR‐21 and miR‐22	[79]
Lentiviral transduction	miR‐181a mimic	MSCs	miR‐181a	[35]
Scaffold‐RBP‐RNA fusion	CD9‐HuR‐	HEK293T cells	(AREs)miR‐155	[36]
Scaffold‐RBP‐RNA fusion	Lamp2a‐TAT‐	HEK293T cells	(TAR)pre‐miR‐199a	[37]
EV sorting motif	CGGGAG	Brown adipocytes	(CGGGAG)miR‐34c	[17]
Sorting RBP overexpression	hnRNPA2B1	Jurkat T cells	miR‐198	[16]
Sorting RBP overexpression	HNRNPA1	Leukemia K562 cells	miR‐320	[38]
Environmental conditioning	Oxidative stress	Bovine granulosa cells	Nrf2 mRNA	[80]
Plasmid /lentiviral transduction	/	AML12 cells	Ldlr mRNA	[39]
Cellular nanoporation	/	MEFs/ nHDFs /nHDFs	PTEN/ COL1A1/ VEGF‐A mRNA	[81, 82, 83]
Scaffold‐RBP‐RNA fusion	CD63‐MS2‐(TAMEL)	HEK293FT cells	(MS2 stem loop) dTomato reporter mRNA	[40]
Scaffold‐RBP‐RNA fusion	CD63‐L7Ae‐(EXOtic system)	HEK293FT cells	(C/D box) catalase mRNA	[41]
Scaffold‐RBP‐RNA fusion	CD63‐PUFe‐	HEK293FT cells	(motif) OX40L mRNA	[42]
Virus‐like self‐assembly mechanism	Arc capsids	HEK293T cells	(ASU)GFP mRNA‐loaded Arc capsids	[43]
Scaffold‐RBP‐RNA fusion	CD9‐HuR‐	HEK293T cells	(AREs) dCas9 mRNA	[36]
Scaffold‐RBP‐RNA fusion	CD63‐Com‐	HEK293T cells	RNP (com‐sgRNA, Cas9)	[44]
Scaffold‐Cas9 protein fusion	CD63‐intein‐	HEK293T cells	RNP (Cas9, sgRNA)	[26]
Scaffold‐Cas9 protein fusion	BASP1‐	HEK293 cells	Cas9 protein	[45]
Tag‐Cas9 protein fusion	ABI‐	HEK293FT cells	RNP (ABI‐Cas9, sgRNA)	[46]
Tag‐Cas9 protein fusion	Myristoylation epitope	HEK293T cells	RNP (N‐myristoylated Cas9, sgRNA)	[84]
Cleavable Scaffold‐Cas9 protein fusion	CD9‐mMaple3‐ (MAPLEX)	HEK293T cells	RNP (dCas9‐DNMT3A, sgRNA)	[60]

#### 3.2.1 Environmentally Induced and Genetically Mediated miRNA Loading

The transcriptomes of different cell types are partially mirrored in their EV RNA cargo. Further studies have demonstrated that the miRNA composition of EVs can be reprogrammed by altering the culture conditions of the parental cells, thereby enabling functional enrichment of specific miRNAs. For example, hypoxic preconditioning of cardiac progenitor cells (CPCs) led to the enrichment of miR‐15b, miR‐17, miR‐20a, miR‐103, miR‐199a, miR‐210, and miR‐292 in their EVs, which enhanced regenerative potential [78]. Likewise, 3D culture of MSCs not only increased EV yield but also elevated miR‐21 and miR‐22 levels [79]. Environmental modulation of EV miRNA profiles largely depends on the global cellular stress response to external cues. Such responses activate broad transcriptional and post‐transcriptional regulatory networks, leading to multifactorial changes that affect numerous miRNAs simultaneously. Consequently, while these approaches effectively generate functionally enhanced EVs, they lack the precision required for selective enrichment of individual miRNAs. To achieve more controlled and target‐specific loading, researchers have turned to genetic engineering approaches that directly manipulate miRNA expression within parental cells. For instance, lentiviral transduction of MSCs with miR‐181a mimics yielded EVs selectively enriched in this miRNA [35]. Notably, both environmental conditioning and genetic overexpression approaches rely on the cell's native miRNA sorting and secretion pathways, without requiring direct intervention in the EV biogenesis machinery. The resulting miRNAs are therefore encapsulated within the EV lumen, following the same topological fate as endogenous miRNA cargo.

While the above interventions improve miRNA abundance, recent advances have focused on manipulating the sorting machinery that governs miRNA packaging into EVs. Analogous to the strategy of delivering proteins via fusion with EV scaffold proteins, fusing scaffold proteins with RBPs enables the incorporation of RBPs‐associated target miRNAs into EVs. Li et al. implemented this strategy by fusing human antigen R (HuR, an RBP that binds AU‐rich elements (AREs) in RNA 3′UTR) to the C‐terminal intracellular domain of CD9, ensuring that HuR faces the EV lumen [36]. The resulting CD9‐HuR construct efficiently enriched native AREs‐containing miR‐155 into EVs, effectively hijacking the endogenous HuR‐AREs interaction and repurposing it for EV loading. Functional assays confirmed that the encapsulated miR‐155 was successfully delivered to recipient cells and mediated suppression of its endogenous target genes. Similarly, the HIV‐1 TAT peptide was displayed on EV membranes via a Lamp2a fusion, with the peptide fused to the cytoplasmic C‑terminus of Lamp2a to position it inside the EV lumen [37]. The loop region of pre‑miR‑199a was replaced with the TAR RNA stem‑loop, a high‑affinity TAT ligand. This engineered TAT‐TAR interaction enabled efficient enrichment of modified pre‐miR‐199a into EVs, significantly increasing mature miR‐199a‐3p levels. In both examples, the RBP‐scaffold fusion strategy ensures intraluminal loading of the target miRNA. Critically, to achieve this topology, the RBP or RNA‐binding peptide must be fused to a cytosolic domain of the scaffold, such as the N‐ or C‑terminus of a tetraspanin (e.g., CD9, CD63) or the C‑terminal tail of a type I transmembrane protein (e.g., Lamp2a), so that the RNA‐protein complex is captured on the cytosolic face of the budding ILVs and ultimately deposited within the EV lumen.

In addition, a promising strategy involves harnessing the natural sorting motifs within miRNAs to enrich target miRNAs into EVs. A number of EV‐enriching motifs have been identified, including GGAG [16, 85], GGCU [18], UAGGUA [86], AGAGGG [38], AAUGC [87], UCAGU [88], and CGGGAG [17]. Based on this principle, artificially incorporating potent EV‐enrichment motifs into target miRNAs can significantly boost their loading efficiency. For instance, introducing a strong EV‐enriching motif (e.g., CGGGAG) into a naturally cell‐retained miR‐34c resulted in a several‐fold increase in its EV enrichment in brown adipocytes [17]. Notably, this motif‐based sorting machinery exhibits both universal and cell‐type‐specific features. By systematically profiling EVs derived from five metabolically important cell types (white adipocytes, brown adipocytes, myotubes, hepatocytes, and endothelial cells), Garcia‐Martin et al. revealed that several motifs (e.g., GGAG) are shared across multiple cell types, whereas their enrichment efficacy can vary significantly [17]. Furthermore, individual cell types possess unique motifs that operate almost exclusively in their respective contexts, such as the NGGUNCA motif specific to white adipocytes. This cell‐type‐specific sorting mechanism underscores the critical importance of selecting optimal motifs that are compatible with the EV‐producing cells to maximize loading efficiency.

Moreover, these sorting motifs function through their interaction with specific cellular RBPs. Key RBP‐motif pairs identified to date include hnRNPA2B1 (which recognizes GGAG), [16] SYNCRIP (which recognizes GGCU), [18] hnRNPA1 (which recognizes AGAGGG and UAGGUA), [38, 86] FMR1 (which recognizes AAUGC), [87] YBX1 (which recognizes UCAGU), [88] as well as Alyref and Fus (which recognize CGGGAG) [17]. Consequently, alongside engineering the miRNA cargo itself, the strategic overexpression of these specific RBPs in parental cells presents a complementary and effective strategy to enhance the EV loading of cognate motif‐containing miRNAs. For example, overexpression of hnRNPA2B1 in human Jurkat T cells significantly increased the packaging of GGAG‐containing miR‐198 into EVs [16]. Similarly, overexpression of HNRNPA1 in chronic myeloid leukemia K562 cells promoted the enrichment of miR‐320, which harbors an AGAGGG motif [38]. In summary, the rational design of miRNA sequences coupled with the modulation of the expression of these RBPs in parental cells constitutes a powerful dual‐pronged approach for enhancing the targeted loading of functional miRNAs into EVs. These approaches, adding EV‐enrichment motifs to the miRNA or overexpressing cognate sorting RBPs, amplify the cell's endogenous sorting pathways rather than introducing exogenous targeting machinery. The miRNAs loaded via these native mechanisms are, like their unmodified counterparts, localized within the protective environment of the EV lumen.

#### 3.2.2 RBPs‐Mediated and Virus‐Inspired mRNA Loading

While reports are relatively scarce, environmental stimuli have also been shown to influence mRNA expression within EVs. For instance, exposure of bovine granulosa cells to hydrogen peroxide‐induced oxidative stress markedly altered the mRNA profile of their released EVs. Compared with EVs derived from unstressed cells, those produced under oxidative stress exhibited significant upregulation of the transcription factor Nrf2 and its downstream antioxidant genes CAT and TXN1 [80]. In addition to environmental stimuli, plasmid or viral transfection represents a more direct approach to engineer the mRNA cargo of EVs. For instance, Li et al. successfully loaded Ldlr mRNA into EVs secreted by AML12 hepatocytes by transfecting the cells with either a plasmid or a lentiviral vector [39]. Administration of these engineered EVs effectively restored LDLR protein function in Ldlr‐deficient mice, reversing hypercholesterolemia and mitigating atherosclerosis. Moreover, cellular nanoporation enables large‐scale production of mRNA‐enriched EVs by transiently permeabilizing the plasma membrane with controlled electrical pulses, which enhances both EV secretion and mRNA packaging efficiency. The resulting EVs have demonstrated therapeutic efficacy in diverse disease models, such as those loaded with PTEN mRNA restoring tumor suppressor activity in PTEN‐deficient glioma [81], those encapsulating COL1A1 mRNA promoting collagen regeneration and reducing wrinkle formation in photoaged skin [82], and those delivered with VEGF‐A mRNA enhancing angiogenesis and improving cardiac function in myocardial infarction (MI) [83]. Additionally, Nawaz et al. used lipid nanoparticles (LNPs) single‐guide RNA to deliver VEGF mRNA into CPCs, obtaining EVs naturally loaded with this mRNA [89]. These CPC‑derived EVs achieved the highest efficiency in promoting angiogenesis per amount of VEGF‑A protein produced in vitro, and caused minimal inflammatory cytokine production in cardiac tissue upon intramyocardial injection in vivo. Whereas environmental stress broadly remodels the cellular transcriptome, nanoporation, transient transfection, and LNP‑mediated delivery enable the selective overexpression of a specific mRNA of interest. The elevated cytosolic concentration of this mRNA then drives its passive incorporation into EVs via the natural ILV biogenesis pathway, without requiring manipulation of the sorting machinery.

**FIGURE 5:**
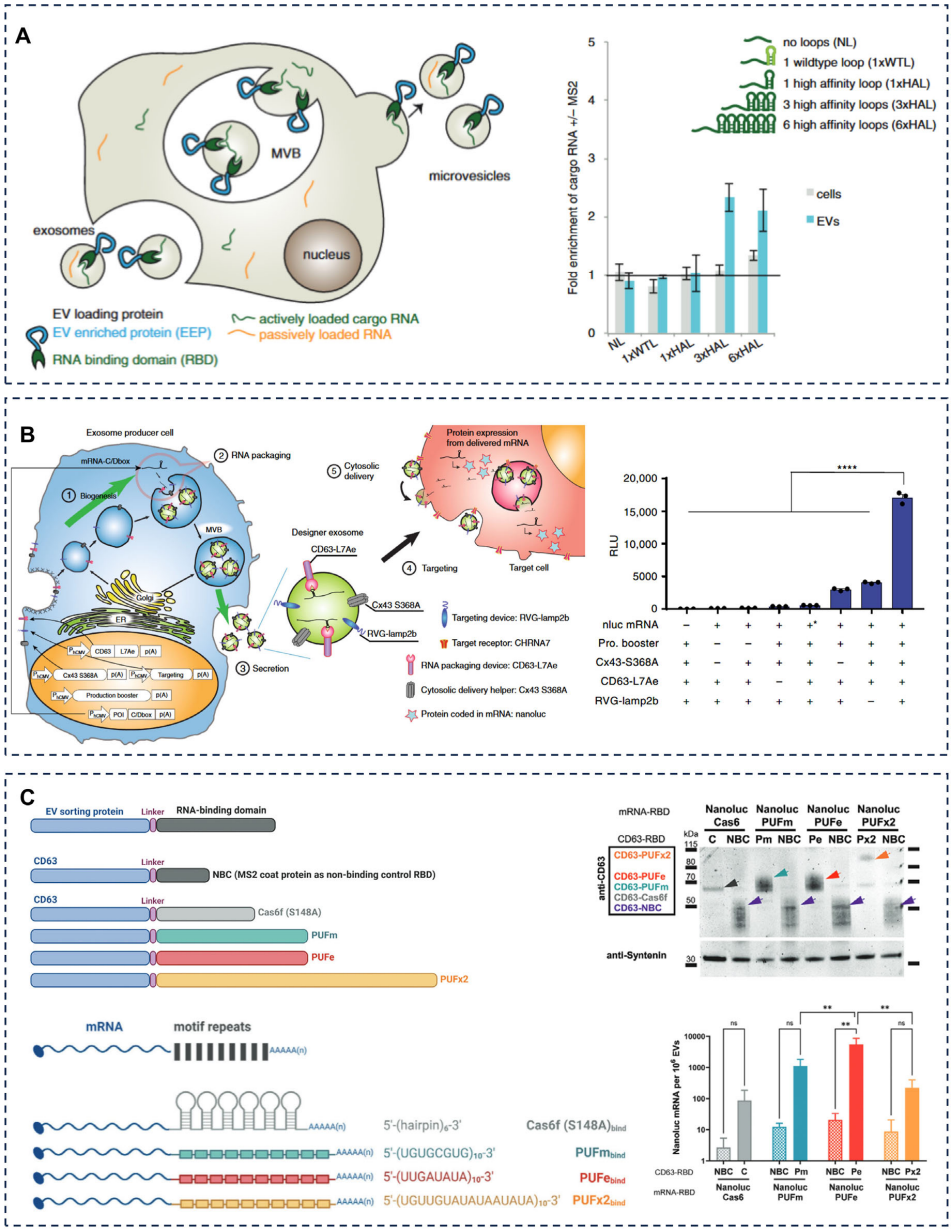
RBPs‐mediated mRNA loading strategies. (A) Schematic illustration of the design and application of the TAMEL platform for mRNA loading into EVs. The right panel shows that cargo RNAs containing three or six high‐affinity MS2 loops (3×HAL, 6×HAL) achieve robust enrichment. Reproduced with permission [40]. Copyright 2016, Wiley. (B) Schematic illustration of the design and application of the EXOtic system for mRNA loading. The right panel evaluates the necessity of key components within the EXOtic system. HEK‐293T producer cells were transfected with plasmids encoding the nluc reporter bearing a C/D box motif together with a full or partial set of EXOtic components. The supernatant containing the resulting engineered EVs was applied to target cells, and nluc activity was assayed. Reproduced with permission [41]. Copyright 2018, by Springer Nature. (C) Schematic illustration of the design and application of the CD63‐PUFe loading system. CD63 was fused to novel RNA‐binding domains (RBDs; Cas6f, PUFm, PUFe, or PUFx2), while target mRNA was codon‐optimized and contained 6–10 RBD‐specific motif repeats in its 3' UTR. The right panel displays functional validation, including western blot detection of the CD63‐PUFe scaffold in EVs and RT‐qPCR‐based absolute quantification demonstrating high‐efficiency enrichment of target mRNAs. Reproduced with permission [42]. Copyright 2024, Wiley‐VCH GmbH.

The strategic fusion of mRNA with RBPs has led to the development of an active loading system characterized by a “scaffold protein‐RBPs‐specific sequence” architecture, which significantly enhances the enrichment of mRNA within EVs. This approach involves fusing an EV scaffold protein with a specific RBP, thereby localizing the RBP to the EVs. Concurrently, the corresponding RBPs recognition sequence is engineered into the untranslated region of the target mRNA. This design facilitates the specific binding between the RBPs and the mRNA during EV biogenesis, enabling efficient packaging of the mRNA into the EVs. For instance, Hung et al. established the Targeted and Modular EV Loading (TAMEL) platform (Figure 5A) [40]. This system employs the MS2 bacteriophage coat protein as the RBP, which recognizes and binds to the MS2 stem‐loop structure embedded in the 3'UTR of the target mRNA. Using HEK293FT producer cells, they successfully demonstrated the efficient loading and delivery of a dTomato reporter mRNA, establishing the core principles of this platform. Similarly, the EXOtic (exosomal transfer into cells) system utilizes the archaeal ribosomal protein L7Ae as the RBP, which specifically interacts with the C/D box RNA motif in the mRNA (Figure 5B) [41]. Also utilizing HEK293FT‐derived EVs, the EXOtic system delivered catalase mRNA to the brain, where it mitigated neuroinflammation and neurotoxicity in a model of Parkinson's disease (PD). Furthermore, Zickler et al. systematically compared several engineered RBPs, including the PUF domain variants PUFm, PUFe, and PUFx2, for their loading efficiency (Figure 5C) [42]. Among these, PUFe was identified as the most effective RBP, exhibiting high‐affinity binding to a specific 8‐nucleotide motif. Using HEK293FT cells as EV producers, the study demonstrated that EVs loaded with the immunostimulatory molecule OX40L mRNA could elicit a potent antitumor immune response in an aggressive melanoma model. Analogous to the miRNA RBP‐scaffold strategy, this approach relies on the fusion of an RBP to an EV‐enriched scaffold protein. To ensure that the captured mRNA is ultimately encapsulated within the EV lumen, the RBP must be fused to a cytosolic domain of the scaffold.

In addition, Gu et al. employed a distinct strategy by harnessing an endogenous, virus‐like self‐assembly mechanism for mRNA packaging [43]. They engineered leukocytes to produce EVs incorporating retrovirus‐like capsids formed by the self‐assembly of the protein Arc. The Arc capsid intrinsically facilitates the encapsulation of mRNA into EVs. To enhance the stability and efficiency of this system, the researchers introduced the Arc 5' UTR as a key RNA stabilizer, which works synergistically with the Arc protein to significantly boost mRNA loading within leukocyte‐derived EVs.

#### 3.2.3 Scaffold‐ or Tag‐Mediated Encapsulation of Cas9 RNP

Beyond leveraging the natural bioactive cargo of EVs, their potential as delivery vehicles has been extended to engineered functional RNP. A key functional entity within this system is the Cas9 RNP, which consists of Cas9 protein and single‐guide RNA (sgRNA). The Cas9 RNP is highly favored for gene editing due to its high efficiency and reduced off‐target effects [84, 90]. This RNP system is effectively shielded by the EV lipid bilayer, which preserves its structural and functional integrity, while its transient delivery as a preformed complex circumvents risks associated with exogenous DNA integration [90]. Consequently, numerous strategies have been developed to load Cas9 RNP into EVs, which can be broadly grouped into two categories: those that harness RBPs to recruit the complex by engineering the RNA component as a molecular handle, and those that rely on direct protein–protein interactions to recruit the Cas9 protein itself.

Strategies that rely on RNA‐mediated recruitment achieve RNP loading by engineering mRNA or sgRNA molecules with RBPs‐recognition motifs, enabling their selective incorporation into EVs. For instance, Li et al. engineered the dCas9 mRNA to include three AREs in its 3' UTR, enabling its active enrichment into EVs through direct binding to the CD9‐HuR fusion protein [36]. Upon delivery to recipient adipogenic stem cells and mouse liver in vivo, EVs loaded with both dCas9‐AREs mRNA and sgRNA targeting the C/ebpα promoter demonstrated the successful intracellular translation and assembly of a functional dCas9 RNP complex, mediating significant repression of endogenous C/ebpα expression. In addition, Yao et al. employed a specific Com/com interaction system, where the “com” aptamer was inserted into the sgRNA and the Com protein was fused to CD63, to actively load pre‐assembled Cas9 RNP into EVs [44]. This approach enabled successful in vivo genome editing, as evidenced by a 0.2% indel rate and the restoration of dystrophin expression observed after intramuscular injection into a Duchenne muscular dystrophy mouse model.

In contrast, strategies based on protein–protein interactions directly tether the Cas9 protein to the EV membrane by fusing it to an EV‐enriched scaffold protein. For instance, Liang et al. adapted the CD63‐intein‐cargo system to generate a CD63‐intein‐Cas9 fusion, enabling encapsulation of preassembled Cas9 RNP into EVs [26]. The pH‐sensitive intein facilitated controlled release of Cas9 in the EV lumen, resulting in up to 80% editing efficiency in recipient cells. Similarly, Dooley et al. employed the novel scaffold protein BASP1 by fusing Cas9 to its C‐terminal fragment, providing a crucial vehicle for the potential delivery of Cas9‐based therapeutics [45]. Recruitment tag‐guided endogenous loading provides another effective mechanism. For example, fusion of the plant‐derived abscisic acid Insensitive (ABI) domain to the C‐terminus of Cas9 created a Cas9‐ABI construct [46]. The ABI domain functioned as a potent loading tag, significantly enriching the Cas9 protein into EVs during their biogenesis. EVs loaded with Cas9‐ABI RNP mediated highly efficient editing at the CXCR4 locus in primary human CD4^+^ T cells. Additionally, Whitley et al. developed a myristoylation‐based strategy by fusing an Src kinase‐derived N‐terminal octapeptide to Cas9, promoting its packaging along with sgRNA into EVs [84]. Notably, Han et al. employed the MAPLEX platform to deliver a fusion protein comprising catalytically dCas9 and the catalytic domain of DNA methyltransferase DNMT3A [60]. This sgBace1‐dCas9‐DNMT3A system was designed not to cleave DNA but to target the Bace1 promoter, relevant in Alzheimer's disease (AD), and induce locus‐specific DNA methylation. This CRISPR‐based epigenome editing approach led to sustained Bace1 suppression, improved cognitive deficits, and reduced amyloid pathology in an AD mouse model.

In the strategies described above, Cas9 RNP loading is achieved by tethering either the RNA component (via RBP‐scaffold fusions) or the Cas9 protein itself (via direct scaffold fusion or peptide tags) to the cytosolic face of the endosomal membrane. This tethering routes the cargo into ILVs during MVB formation. Consequently, the molecular nature of the encapsulated cargo varies across strategies: some approaches load only mRNA, which is translated and assembled into a functional RNP in the recipient cell; others load pre‐assembled Cas9 RNP as a soluble complex within the lumen; and still others load fusion proteins where Cas9 remains covalently linked to the scaffold or tag, requiring subsequent release or processing to become fully functional.

## 4 Applications of Engineered EVs in Multiple Diseases

Advances in EV bioengineering have greatly improved their therapeutic efficacy by enabling precise cargo loading, enhanced targeting, and prolonged activity. Recent publications revealed that engineered EVs for systemic injection are primarily applied in cardiovascular, hepatic, neurological, and oncological diseases, where engineering strategies are designed to achieve two primary purposes: cargo loading and delivery enhancement. Given that engineering purposes for EVs are inherently shaped by disease pathophysiology and therapeutic mechanisms, this section classifies the therapeutic applications of engineered EVs according to specific disease categories. Within each category, the discussion is organized to follow the sequence of engineering strategies previously described, while further distinguishing between the different types of loaded cargo and specifying the experimental validation level (Table 3).

**TABLE 3:** Engineered EVs in disease therapy.

Diseases	Loading strategy	Detail of methods	Cargos	Engineering purpose	Model type	Ref.
**Cardiovascular disease**						
MI‐induced cardiac injury	Environmental conditioning	Culture ADSCs with endothelial differentiation medium	miR‐31	Cargo loading	MI mouse model	[91]
		Hypoxic preconditioning of CPCs	miR‐15b, miR‐17, miR‐20a, et al.	Cargo loading	Rat model of ischemia reperfusion	[78]
		Stimulate EPCs with silicate ions	miR‐126a‐3p	Cargo loading	MI mouse model	[51]
		Transfect MSCs with miR‐181a	miR‐181a	Cargo loading	Mouse model of ischemia reperfusion	[35]
	Overexpression in parental cells	Overexpress the βARKct inhibitory peptide in CDCs	βARKct inhibitory peptide	Cargo loading	Acute MI and high‐dose isoproterenol injury mouse model	[92]
	Cargo‐scaffold fusion	Transfect MSC with the IMTP fused with Lamp2b	IMTP	Delivery	MI mouse model	[93]
		Express Lamp2b‐HHP fusion protein in CDC‐EVs	HHP	Delivery	Transverse aortic constriction mouse model	[94]
		Express Lamp2b‐CMP fusion protein in CDC‐EVs	CMP	Delivery	Wild‐type mouse	[95]
Atherosclerosis	Environmental conditioning	Laminar shear stress induced HUVEC	miR‐34c‐5p	Delivery	ApoE^−/−^ mouse atherosclerotic model	[96]
**Hepatic disease**						
ALI	Environmental conditioning	Heat shock pretreatment of MSCs	Proteins and miRNAs with anti‐inflammatory and antioxidant stress effects	Cargo loading	Hepatic ischemia‐reperfusion injury and acetaminophen‐induced liver injury in a mouse model	[50]
		IL‐6 stimulation of hUC‐MSCs	miR – 455 – 3p	Cargo loading	Chemically induced ALI mouse model	[97]
	Overexpression in parental cells	Overexpressing CD47 in bEnd.3 cells	CD47	Delivery	Drug‐induced ALI mouse model	[98]
Liver fibrosis	Overexpression in parental cells	Transfect circCDK13 into 293T cells	circCDK13	Cargo loading	Liver fibrosis mouse model	[99]
	Cargo‐scaffold fusion	Transduced HSTP1‐Lamp2b fusion protein into hUC‐MSCs	aHSCs‐targeted peptide	Delivery	Liver fibrosis mouse model	[100]
**Neurological disorders**						
SCI	Environmental conditioning	3D MSC culture	Increases the quantity and quality of EVs	Cargo loading	SCI rat model	[101]
PD	Scaffold‐RBP‐RNA fusion	Engineering HEK‐293T cells with the CD63‐L7Ae RNA‐binding module	Catalase mRNA	Cargo loading	6‐OHDA‐induced PD mouse model	[41]
Delivery to the mouse brain	Cargo‐scaffold fusion	E‐NoMi platform	Cre recombinase	Cargo loading	Wild‐type mouse	[71]
	Cargo‐scaffold fusion	Express Lamp2b‐RVG peptide fused protein in dendritic cells	RVG peptide	Delivery	Wild‐type mouse	[53]
Neuro‐inflammation	Virus‐like self‐assembly mechanism	Arc/Gag‐like scaffold self‐assembly in leukocyte EVs for mRNA loading.	Cre recombinase mRNA	Cargo loading	Naturally senescing mouse	[43]
**Cancer**						
Melanoma	Cargo‐scaffold fusion	IL‐2‐PDGFR transmembrane fusion in Jurkat T cells for autocrine signaling.	IL‐2	Cargo loading	Syngeneic cancer and xenograft mouse models	[102]
	Scaffold‐RBP‐RNA fusion	CD63‐PUFe/VSV‐G engineered HEK293T EVs for motif‐specific mRNA loading.	OX40L mRNA	Cargo loading	B16F10 melanoma mouse model	[42]
		Express anti‐CD2 (scFvs) ‐PDGFR transmembrane domain fusion protein	Cas9/sgRNA complexes	Cargo loading	Primary human CD4^+^ T cells	[46]
Melanoma	Cargo‐scaffold fusion	CD63‐Z‐domain engineered Fc‐EVs for modular, high‐density IgG antibody‐guided targeting.	PD‐L1 or HER2 antibodies	Delivery	Melanoma mouse model	[103]
Solid tumor	Cargo‐scaffold fusion	Anchored PH20 to HEK293T exosome membranes via a GPI anchor	PH20	Delivery	Subcutaneous Xenograft Mouse Model	[104]
Primary carcinoma	Environmental conditioning	MGC803/HepG2 EVs via pH 4.0 acidic culture	Lipid‐reprogrammed	Delivery	PDX tumor mouse model	[105]
Multi cancers	Cargo‐scaffold fusion	DR5‐scFv‐PDGFR‐engineered NK92‐EVs for targeted apoptosis of tumor and stromal cells	DR5‐scFv	Delivery	Melanoma, liver, and breast cancer mouse models	[68]
Tumors with different surface antigens	Overexpression in parental cells	Bispecific anti‐CD19 scFv/PD‐1 engineered DC‐EVs	anti‐CD19 scFv and PD‐1	Delivery	Subcutaneous colorectal carcinoma mouse model	[106]

Notably, while the strategies elaborated herein primarily emphasize endogenous engineering, some featured cases incorporate exogenous modifications, constituting a “hybrid strategy”. However, for these hybrid strategies, our discussion focuses specifically on the therapeutic functional improvements and biological fidelity attributed to the endogenous components, which, as previously noted, better preserve the structural integrity and inherent bio‐activity of the vesicles.

### 4.1 Cardiovascular Disease

Cardiovascular disease (CVD) remains the leading cause of morbidity and mortality worldwide. Among CVDs, MI is the most common and devastating manifestation. MI occurs when coronary artery occlusion leads to insufficient blood supply and widespread cardiomyocyte death. The release of damage‐associated signals triggers a strong inflammatory response, in which infiltrating macrophages initially exacerbate injury but later contribute to tissue repair. Endothelial cell dysfunction further impairs angiogenesis, while activated fibroblasts deposit excessive extracellular matrix (ECM), driving fibrosis and ventricular stiffening [107]. Over time, these maladaptive processes culminate in adverse ventricular remodeling and hypertrophy, ultimately progressing to heart failure (HF). Because these pathological processes involve hypoxia, oxidative stress, and chronic inflammation, researchers have focused on simulating these conditions, such as through hypoxic, oxidative, or inflammatory stimuli, to enhance the therapeutic potential of EVs. EVs isolated from MSCs, CPCs, pluripotent stem cell‐derived cells, and differentiated somatic cells have shown promise in preclinical models, while endogenous engineering can further optimize their therapeutic efficacy against CVD [108]. The primary therapeutic challenge in CVD lies in the continuous mechanical movement of the heart and vasculature, which makes it difficult for systemically administered agents to reach and maintain an effective local concentration. In this context, the functional priority of endogenous engineering focuses on enhancing targeting capabilities to overcome these physiological hurdles. Current efforts are centered on “spatiotemporal precision,” modifying EV surfaces with myocardial‐specific or damaged tissue‐specific peptides (such as ischemic myocardium‐targeting peptide (IMTP) or cardiomyocyte‐binding peptide (CMP)) to address the inherently poor accumulation of EVs in cardiac tissue. This shift from “passive diffusion” to “active intervention” ensures lesion homing and long‐term retention even under severe hemodynamic stress. By integrating these bioengineered EVs with targeted delivery, researchers can more effectively address multifactorial CVD pathology, providing a precision approach to vascular repair and functional recovery.

**FIGURE 6:**
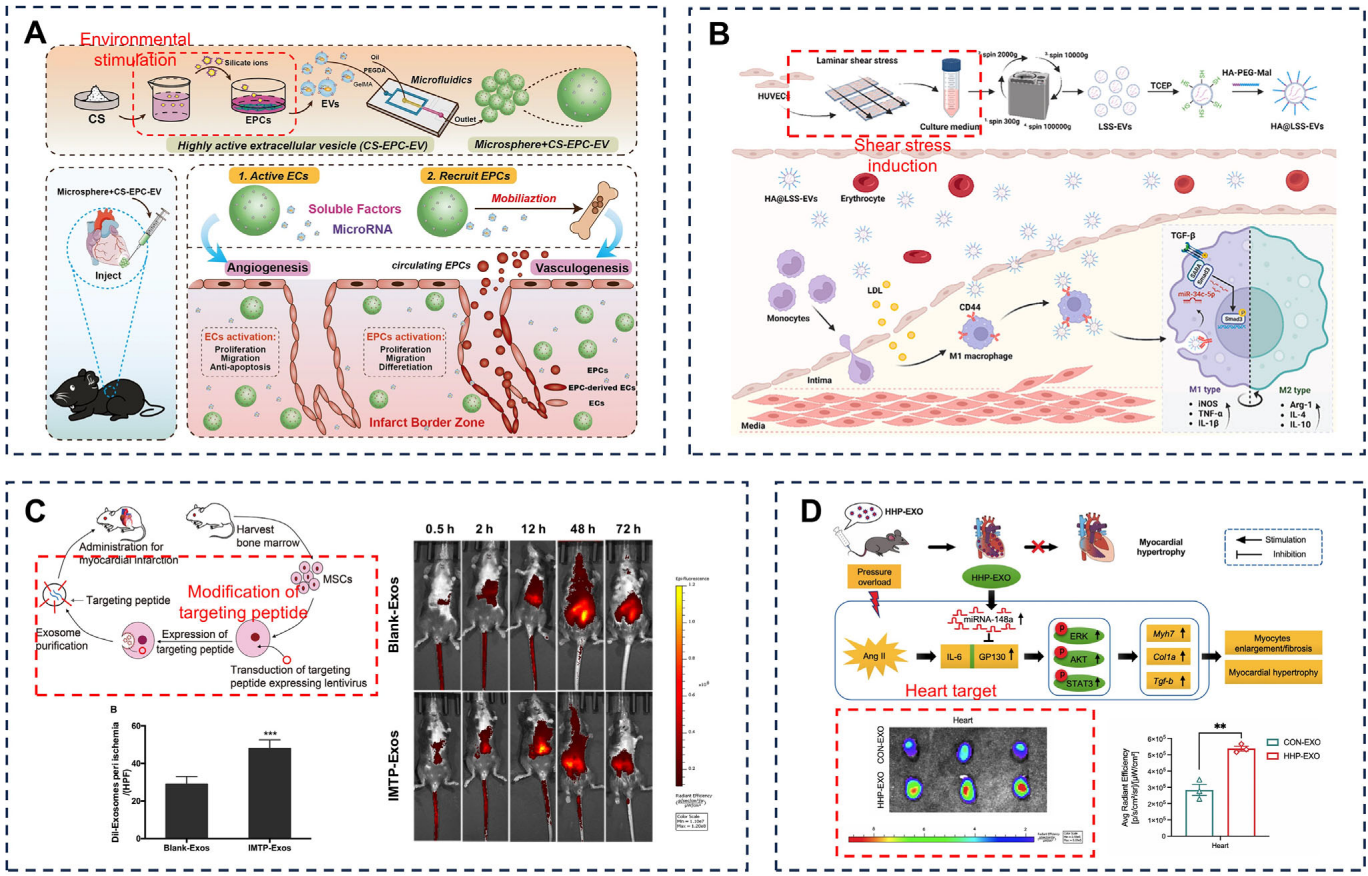
Various engineered EVs for CVD treatment. (A) Schematic illustrating the stimulation of EPCs with silicate ions, leading to the enrichment of EVs with miR‐126a‐3p. These EVs are encapsulated in microspheres using microfluidic technology for controlled release. The microsphere‐loaded EVs are injected into infarcted myocardial tissue, promoting angiogenesis, vasculogenesis, and recovery after MI by stimulating new blood vessel formation, improving myocardial function, and supporting vascular repair. Reproduced with permission [51]. Copyright 2023, Springer Nature. (B) Schematic showing HUVECs exposed to laminar shear stress, inducing the release of EVs rich in miR‐34c‐5p, which reprogram macrophages from a pro‐inflammatory to a reparative phenotype, improving outcomes in atherosclerosis. The LSS‐EVs are further functionalized with HA‐PEG via click‐chemistry conjugation, enabling stable surface display that targets CD44 on inflammatory macrophages, enhancing EV accumulation in atherosclerotic plaques, and promoting more efficient macrophage reprogramming at disease sites. Reproduced with permission [96]. Copyright 2025, Elsevier. (C) Schematic of MSC‐derived EVs fused with the ischemic myocardium‐targeting peptide (IMTP), promoting targeted delivery to infarcted myocardium and enhancing tissue repair. Reproduced with permission [93]. Copyright 2018, Wiley‐VCH GmbH. (D) Schematic of CDC‐derived EVs engineered with CRPPR peptide, which targets neuropilin‐1 on cardiac endothelial cells, improving heart retention and attenuating hypertrophy through miR‐148a‐mediated inhibition of GP130/STAT3/ERK/AKT signaling. Reproduced with permission [94]. Copyright 2022, Springer Nature.

Beyond the intrinsic reparative capacity of stem‐ and progenitor cell‐derived EVs, their therapeutic efficacy can be markedly augmented by environmentally conditioning the producer cells prior to EV release. For example, EVs derived from adipose‐derived stem cells (ADSCs) have been shown to reduce cardiomyocyte apoptosis, stimulate angiogenesis, improve left ventricular ejection fraction, and alleviate MI‐induced fibrosis [109]. When ADSCs were cultured in endothelial differentiation medium supplied with proangiogenic factors such as VEGF, bFGF, and IGF, their EVs became enriched in miR‐31, which augmented endothelial migration and vascular network formation through inhibition of HIF‐1α [91]. Likewise, hypoxic preconditioning of CPCs, mimicking the ischemic myocardial environment, generated EVs enriched in distinct miRNA clusters (e.g., miR‐15b, miR‐17, miR‐20a, miR‐199a, and miR‐210) that promoted angiogenesis, suppressed pro‐fibrotic signaling, and facilitated cardiac recovery [78]. Chemical cues, such as silicate ions, were effective in enhancing the therapeutic potential of EPCs. When treated with silicate ions, EPCs secreted EVs (CS‐EPC‐EVs) enriched with miR‐126a‐3p. These EVs were then encapsulated into microspheres using microfluidic technology, which allows for controlled release. Microsphere‐loaded CS‐EPC‐EVs were injected directly into the infarcted myocardial tissue of mice, resulting in significant therapeutic effects. The treatment promoted revascularization and functional recovery by stimulating the formation of new blood vessels and improving the function of the damaged myocardium. The microsphere delivery system not only protects the EVs from degradation but also enhances the local concentration of miR‐126a‐3p, which plays a crucial role in endothelial cell function and angiogenesis. Additionally, the EVs recruited EPCs from the bloodstream to the infarcted area, where they differentiated and integrated into the damaged blood vessels, thereby supporting vascular repair and contributing to the overall recovery of heart tissue after MI (Figure 6A) [51]. Extending this concept to physiologically relevant conditions, exposure of endothelial cells to laminar shear stress induced HUVEC EV (LSS‐EVs) rich in miR‐34c‐5p, a key regulator that shifts macrophages from a pro‐inflammatory M1 state toward a reparative M2 phenotype through modulation of the TGF‐β/Smad3 signaling axis. HUVECs are particularly suitable for generating these vesicles because they represent the primary endothelial population exposed to flow forces in the vascular system, ensuring that the EV cargo reflects physiologically protective shear‐dependent cues. These LSS‐EVs shift macrophages from a pro‐inflammatory M1 state toward a reparative M2 phenotype through modulation of the TGF‐β/Smad3 signaling axis. By leveraging this intrinsic immunoregulatory cargo, LSS‐EVs achieve efficient macrophage reprogramming at disease sites, ultimately dampening vascular inflammation and improving pathological outcomes in atherosclerosis (Figure 6B) [96].

Collectively, these environment‐mimicking strategies demonstrate that preconditioning with disease‐relevant cues (growth factors, hypoxia, bioactive ions, or shear stress) augments EV therapeutic potency through enhanced yield (up to five fold), selective miRNA enrichment, and functional reprogramming of recipient cells. However, key barriers remain: upstream signaling pathways linking stimuli to EV cargo changes are incompletely defined; scalable, Good Manufacturing Practice (GMP)‐compatible preconditioning workflows are lacking; long‐term safety of systemically delivering enriched miRNAs, particularly those with known oncogenic potential, requires rigorous evaluation; and standardized quality metrics for cargo loading and residual stimulatory factors are absent. Addressing these gaps through mechanistic dissection, manufacturing innovation, and comprehensive preclinical safety profiling will be essential for clinical translation.

In addition to environmental modulation, genetic engineering of EV‐producing cells offers a powerful strategy to address the key pathological processes of inflammation and cardiomyocyte apoptosis in MI and HF. For instance, transfection of MSCs with miR‐181a produced EVs that promoted reparative polarization of peripheral blood mononuclear cells and markedly improved left ventricular ejection fraction in ischemia/reperfusion models [35]. Moreover, since GRK2 upregulation and subsequent β‐adrenergic receptor desensitization are key drivers of maladaptive remodeling in MI and HF, Kwon et al. engineered cardiac‐derived cells (CDCs) to overexpress the βARKct inhibitory peptide. Specifically, βARKct‐CDC EVs exhibited enhanced anti‐apoptotic and immunomodulatory effects, including a reduction in neutrophil infiltration and significantly decreased the mRNA levels of pro‐inflammatory cytokines IL‐6 and MCP‐1 in myocardial tissue, while increasing the anti‐inflammatory cytokine IL‐10 in the bloodstream, thereby providing marked cardioprotection in both MI and HF models [92]. Beyond direct genetic modification, an alternative engineering paradigm involves “indirectly reprogramming” the EV cargo by first delivering therapeutic mRNA to producer cells via lipid nanoparticles (LNPs), which is subsequently packaged into secreted EVs. Nawaz et al. demonstrated this concept by treating CPCs with LNP‐encapsulated VEGF‐A mRNA, and then harvesting the CPC‐derived EVs. These EVs not only delivered functional VEGF‐A mRNA that promoted angiogenesis in vitro and in vivo, but also carried multiple upregulated proangiogenic transcripts induced by the LNP treatment, achieving superior vascular network formation per unit of VEGF‐A protein produced compared to LNPs alone. Critically, when injected intramyocardially, these CPC‐EVs induced markedly lower expression of inflammatory cytokines (including IL‐6 and TNF‐α) in cardiac tissue than LNPs, naked mRNA, or EVs from non‐cardiac cell sources, highlighting a cell‐type‐specific immunomodulatory advantage [89]. However, both engineering strategies share common limitations: miR‐181a overexpression relies on lentiviral vectors with insertional mutagenesis risk and lacks long‐term safety assessment; βARKct‑EVs exhibit model‑specific efficacy (pronounced in catecholamine toxicity but not in conventional MI), and neither approach addresses low EV yield, unclear in vivo targeting efficiency, or scalability hurdles. Future efforts should focus on non‑viral cargo loading, cardiac‑targeting EV modifications, GMP‑compliant large‑scale production, and rigorous large‑animal long‑term safety and dose‑response studies to facilitate clinical translation.

To further enhance therapeutic specificity, targeting EVs to specific cardiac tissues addresses localized pathology, such as ischemic injury or fibrosis, to promote more effective tissue repair. One commonly used strategy is to genetically fuse targeting peptides to Lamp2b. In this design, parental cells were engineered to express Lamp2b‐peptide fusion proteins, in which the peptide was linked to the extracellular N‐terminus of Lamp2b, ensuring that the targeting motifs are displayed on the EV surface during biogenesis [12]. For instance, EVs derived from MSCs, which are selected for their high secretion capacity and intrinsic cardioprotective properties, were engineered by fusing Lamp2b with the IMTP through molecular cloning and lentiviral packaging. After intravenous injection, these IMTP‐EVs gradually accumulated in the infarcted myocardium, with clear fluorescent signals appearing at 12 h, peaking at 48 h, and remaining detectable up to 72 h, while off‐target accumulation in liver, spleen, lung, and kidney was minimal. This efficient targeting enhanced therapeutic effects, including reduced apoptosis and fibrosis, attenuated inflammation, and increased angiogenesis in the ischemic region (Figure 6C) [93]. Similarly, leveraging the strong cardioprotective and pro‐regenerative capacity of CDCs, the homing peptide CRPPR (HHP), which binds neuropilin‐1 on cardiac endothelial cells, has been incorporated into CDC‐EVs. This is achieved by genetically fusing HHP to Lamp2b in CDCs, enabling stable surface display of the peptide on secreted EVs. After intravenous administration, HHP‐engineered CDC‐EVs exhibit markedly enhanced cardiac tropism, showing approximately doubled cardiac retention compared with unmodified CDC‐EVs while maintaining negligible uptake in non‐cardiac organs. This improvement enabled more efficient delivery of miR‐148a to cardiomyocytes, thereby attenuating pathological hypertrophy through inhibition of the GP130/STAT3/ERK/AKT signaling pathway (Figure 6D) [94]. Likewise, fusion with a CMP enabled EVs from CDCs to recognize cardiomyocyte surface protein tenascin‐X, thereby improving cardiac retention and reducing apoptosis [95]. Despite these successes, the Lamp2b‑based targeting strategies share several limitations. The cognate receptors for IMTP and CMP remain unidentified, and although HHP is known to bind neuropilin‑1, the exact binding affinities and specificities have not been quantitatively characterized. Intravenously injected targeted EVs still undergo substantial hepatic and splenic clearance, which limits the fraction reaching the heart. Long‑term safety (e.g., chronic inflammation, off‑target effects on non‑cardiomyocytes) has not been evaluated. As a potential solution, in situ delivery methods, such as cardiac microneedle‐mediated administration, have emerged to maximize targeting precision by delivering EVs directly to the site of injury. However, the path to clinical translation for such localized strategies is complicated by significant challenges that exceed those of systemic routes, such as potential tissue damage and the requirement for sophisticated interventional guidance. Future improvements should focus on the identification of the molecular targets and large‑animal studies with extended follow‑up to validate both efficacy and safety.

### 4.2 Liver Disease

Liver diseases can be broadly classified into acute forms, such as acute liver injury (ALI) and acute liver failure (ALF), and chronic conditions, such as liver fibrosis. Together, they encompass a heterogeneous spectrum of pathological processes with high morbidity and mortality. Although liver transplantation remains the gold‐standard treatment, its application is constrained by donor organ shortage and the need for lifelong immunosuppression [110]. These limitations highlight the urgent demand for alternative therapeutic strategies. ALI, commonly induced by drugs, toxins, alcohol, or viral infection, is characterized by widespread hepatocyte death, oxidative stress, and inflammatory cell infiltration. Activated macrophages amplify tissue injury by releasing pro‐inflammatory cytokines, and in severe cases, this cascade progresses to ALF, accompanied by systemic complications [111]. Notably, functional impairment of liver sinusoidal endothelial cells (LSECs) during acute injury weakens their ability to secrete protective angiocrine factors such as Wnt2 and Wnt9b, thereby diminishing regenerative signaling and delaying liver repair [112]. Moreover, chronic or unresolved injury perpetuates hepatocyte apoptosis and inflammation, leading to the activation of hepatic stellate cells (HSCs). Once activated, HSCs drive ECM deposition, which ultimately results in liver fibrosis [113]. Despite available pharmacological and supportive therapies, options capable of reversing acute injury or preventing fibrotic progression remain scarce. EVs naturally tend to accumulate in the liver following intravenous administration, making them particularly appealing and feasible for liver‐directed therapies [114, 115]. Given this inherent passive liver tropism, the preferred engineering strategy often bypasses complex organ‐level homing modifications, focusing instead on maximizing therapeutic potency through direct cargo functionalization or parental cell preconditioning. Nonetheless, to prevent excessive nonspecific clearance by the mononuclear phagocyte system (e.g., Kupffer cells) and ensure precise therapeutic action, the priority targeting strategy shifts toward cell‐specific targeting, aiming at specific intrahepatic populations such as injured hepatocytes or activated HSCs, depending on the disease context.

**FIGURE 7:**
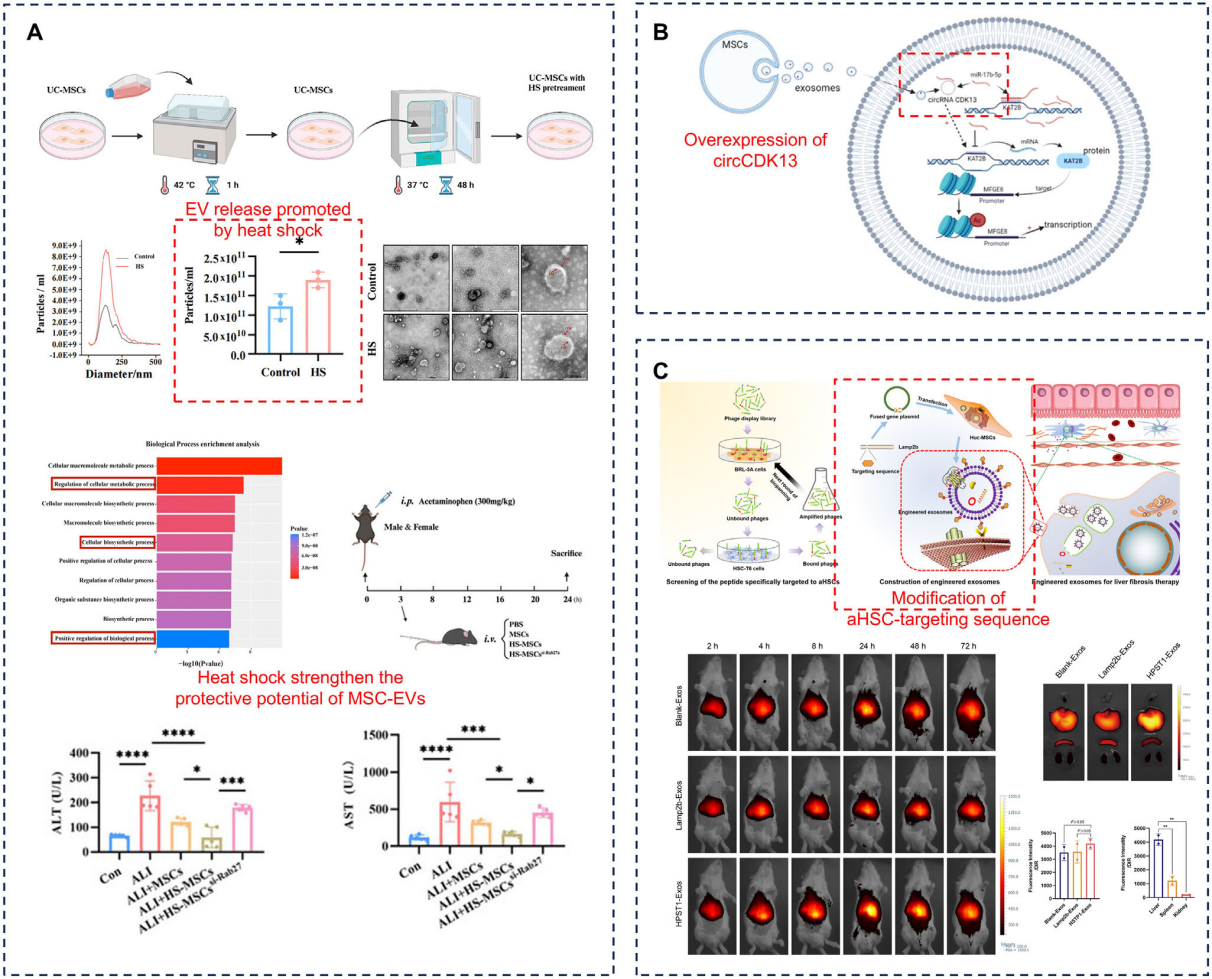
Various engineered EVs for liver disease treatment. (A) Schematic illustrating that heat shock preconditioning (42°C for 1 h) significantly promotes the release of MSC‐EVs by activating the TRPV2‐Ca^2^^+^ signaling pathway, and increases the expression of proteins and non‐coding RNAs associated with anti‐apoptosis, anti‐inflammation, angiogenesis, and antioxidative pathways, thereby enhancing liver protection efficacy. Reproduced with permission [117]. Copyright 2025, Wiley‐VCH GmbH. (B) Schematic illustrating the genetic engineering of MSCs to overexpress therapeutic circular RNAs (e.g., circCDK13), their packaging into EVs, and the subsequent mechanism of action in HSCs. Reproduced with permission [99]. Copyright 2023, Springer Nature. (C) Schematic illustrating the generation of HSC‐targeted EVs by identifying the aHSC‐binding peptide HSTP1 through phage‐display biopanning and displaying this peptide on EVs via fusion with Lamp2b in genetically modified hUC‐MSCs. The resulting HSTP1‐decorated EVs show enhanced uptake by activated HSCs and preferential accumulation in fibrotic regions after systemic administration, leading to improved targeting and anti‐fibrotic activity. Reproduced with permission [100]. Copyright 2022, Springer Nature.

EVs derived from human umbilical cord MSCs (hUC‐MSC‐EVs) exerted hepatoprotective effects through their intrinsic cargo glutathione peroxidase 1, which attenuated oxidative stress and apoptosis in murine models of chemically induced ALF [116]. Beyond intrinsic cargos, environmental and genetic manipulation of parental cells has been employed to enrich EVs with specific therapeutic molecules. For example, heat shock pretreatment of MSCs (42°C for 1 h) significantly enhanced EV release by activating the TRPV2‐Ca^2^^+^ signaling pathway, which triggered YWHAZ succinylation and promoted autophagosome‐multivesicular body fusion (Figure 7A) [117]. The resulting Heat Shock MSC‐EVs (HS‐MSC‐EVs) not only exhibited increased yield but also displayed optimized cargo profiles enriched in proteins and non‐coding RNAs associated with anti‐apoptotic, anti‐inflammatory, pro‐angiogenic, and antioxidative pathways. In hepatic ischemia‐reperfusion injury and acetaminophen‐induced liver injury models, HS‐MSC‐EVs reduced hepatocellular necrosis, neutrophil and macrophage infiltration, and oxidative damage, while enhancing autophagy and tissue repair, thereby conferring superior hepatoprotective efficacy compared to unstressed MSC‐EVs. Similarly, inflammatory priming via IL‐6 stimulation of hUC‐MSCs significantly enhanced single‐cell EV yield by approximately threefold; the resulting EVs were highly enriched in miR‐455‐3p, which effectively suppressed macrophage activation via the PI3K pathway and mitigated liver injury in chemically induced ALI models [97]. EVs also exhibited intrinsic accumulation in the spleen, and considering the spleen's recognized “accomplice” role in LF, our recent work leveraged the hepatic and splenic tropism of EVs [118]. By polarizing macrophages toward an antifibrotic ly6c^low^ phenotype, we generated ly6c^low^ Exo enriched with immunomodulatory proteins such as Sirpa, Anxa1, Cdc42, and Hspd1, and miRNAs including miR‐30e‐5p, miR‐199a‐3p, miR‐143‐3p, and miR‐125b‐5p, which collectively contribute to the regulation of leukocyte differentiation, macrophage activation, and overall immune homeostasis. These vesicles reprogrammed splenic monocytes from a proinflammatory ly6c^high^ phenotype toward an anti‐inflammatory ly6c^low^ state and directly promoted the polarization of hepatic macrophages toward a reparative phenotype, thereby coordinating the liver–spleen immune axis and mitigating fibrosis. While these strategies demonstrate a compelling balance of therapeutic potency and efficiency, they encounter distinct hurdles regarding safety and scalability. Specifically, the high safety profile of conventional EVs can be compromised by biological induction; for instance, the use of stimuli such as LPS or IL‐6 to generate therapeutic EVs carries inherent risks of residual endotoxins or exogenous cytokines. In contrast, physical induction methods‐such as thermal or mechanical stimulation‐offer a more translatable and “cleaner” alternative for GMP‐compliant manufacturing as they eliminate the need for exogenous biological additives. However, ensuring consistent cargo enrichment across different batches remains a challenge due to donor‐dependent cellular responses. Future directions should prioritize the establishment of rigorous quality control standards focused on cargo purity and functional potency.

While these environmental and phenotype‐driven approaches result in a broad, synergistic enrichment of various protein and RNA species, other strategies prioritize the precise loading of individual nucleic acid sequences to orchestrate specific regulatory axes. Several studies have shown that MSCs engineered to overexpress therapeutic circRNAs, such as circDIDO1 or circCDK13, can generate EVs that inhibit HSC activation through distinct miRNA‐dependent mechanisms, thereby reducing fibrosis in the chemically induced ALI model [99, 119]. Specifically, MSCs were transfected with a plasmid encoding the circular RNA, which was then efficiently loaded into EVs during their biogenesis. These circCDK13‐enriched EVs (Exo‐circCDK13) transferred circCDK13 to HSCs, where it sponged miR‐17‐5p to upregulate the histone acetyltransferase KAT2B (Figure 7B). KAT2B promoted H3K27 acetylation and MFGE8 transcription, thereby suppressing TGF‐β1‐induced HSC activation and extracellular matrix deposition. Activation of the circCDK13/miR‐17‐5p/KAT2B axis also attenuated PI3K/AKT and NF‐κB signaling, reducing inflammation and fibrosis in vivo. Both circDIDO1‐EVs and circCDK13‐EVs utilize plasmid‐mediated endogenous loading to target the miR‐141‐3p/PTEN/AKT and miR‐17‐5p/KAT2B/MFGE8 axes, respectively. Among these, circCDK13‐Exo provides the most robust evidence chain by completing in vivo rescue validation in TAA‐induced fibrosis models, whereas the in vivo efficacy of circDIDO1‐Exo has not yet been documented. A critical gap persists in the systematic safety assessment of these technologies, with common concerns including plasmid DNA carryover and interference with intracellular ceRNA networks. To propel small RNA‐focused EV engineering forward, it is critical to develop active enrichment strategies for precise cargo loading, thereby overcoming long‐standing hurdles in efficiency and specificity. Furthermore, success in this field depends on supplementing in vivo evidence and establishing rigorous quality control standards to mitigate risks associated with stimulus residuals and genetic modifications.

In addition, cell type‐specific targeting has been actively explored. Activated HSCs (aHSCs), as central drivers of fibrosis, represented a key therapeutic target. Lin et al. screened a phage‐displayed random peptide library through iterative biopanning against HSC‐T6 cells to identify ligands with preferential binding to aHSCs, ultimately selecting the peptide HSTP1 after verifying its affinity in both cultured cells and fibrotic liver sections (Figure 7C) [100]. To endow EVs with this targeting capability, they genetically fused HSTP1 to the exosomal membrane protein Lamp2b and transduced hUC‐MSCs using lentiviral vectors, enabling the peptide to be displayed on the surface of secreted EVs. The resulting HSTP1‐modified EVs (HSTP1‐Exos) exhibited enhanced internalization by HSCs in vitro, as evidenced by significantly higher uptake efficiency and membrane colocalization compared with unmodified EVs, and showed preferential accumulation in aHSC‐rich fibrotic regions after systemic administration. These EVs effectively suppressed HSC activation, proliferation, and CCL2 secretion, alleviated collagen deposition, and attenuated liver fibrosis more efficiently than unmodified or Lamp2b‐only EVs. Moreover, Du et al. focused on the protective and regenerative roles of LSECs during hepatic injury and accordingly designed LSEC‐targeted engineered EVs [98]. CD47‐overexpressing bEnd.3 cells secreted EVs enriched with surface CD47, which conferred macrophage evasion via the CD47‐SIRPα axis, extending the EVs' circulation half‐life. Additionally, RLTR‐modified EVs demonstrated enhanced liver‐specific accumulation, with significant enrichment in LSECs, effectively targeting hepatic injury sites and improving therapeutic outcomes. In parallel, a LSEC‐homing peptide (RLTRKRGLK) was inserted into the EV membrane to promote selective recognition by Stabilin‐2 receptors on LSECs. The resulting dual‐engineered EVs efficiently delivered Wnt2 mRNA to LSECs, reactivated endothelial Wnt/β‐catenin signaling, and promoted hepatocyte proliferation and liver regeneration in drug‐induced ALI models. Beyond peptide‐based targeting, aHSCs overexpress receptors such as retinol‐binding protein receptors (RBPRs), offering additional entry points for EV engineering. For example, by exploiting vitamin A‐RBPR interactions, BM‐MSCs have been engineered to produce EVs capable of selectively entering aHSCs and suppressing their activation [120]. These cell‐specific targeting strategies offer distinct advantages in delivery efficiency and therapeutic efficacy. For instance, HSTP1‐EVs significantly enhance in situ accumulation in fibrotic regions. By reducing nonspecific clearance and increasing target cell exposure, these methods maximize therapeutic impact. However, safety assessments for such modifications are currently lagging. Potential risks, including lentiviral residue, the immunogenicity of targeting peptides, and the lack of identified target receptors for many ligands, present barriers to rational optimization. Future clinical translation will require rigorous quality control standards for particle display density and residual contaminants, alongside receptor identification and cross‐model validation to ensure both safety and reproducibility.

### 4.3 Neurological Diseases

Neurological diseases, including stroke, spinal cord injury (SCI), AD and PD, represent a growing global health burden, particularly among aging populations. These conditions share common pathological hallmarks such as neuronal apoptosis, neuroinflammation, oxidative stress, and disruption of neural connectivity, which collectively lead to progressive functional decline [121]. Current therapeutic strategies, including pharmacological agents and neuroprotective interventions, are often limited by poor blood–brain barrier (BBB) permeability, rapid drug clearance, and insufficient targeting of diseased neural tissue [122]. Moreover, conventional treatments largely address symptoms rather than repairing damaged neurons or modulating the detrimental inflammatory microenvironment. In contrast, EVs have emerged as a promising next‐generation nanotherapeutic for neurological disorders owing to their intrinsic biocompatibility, ability to cross the BBB, and neurotrophic potential. In addition to systemic administration, intranasal delivery of EVs has recently shown great promise for neurological therapy, enabling noninvasive, direct transport along the olfactory and trigeminal pathways to the brain while minimizing systemic clearance [123]. Most engineering efforts for neurological disorders have focused on two main directions: first, reinforcing the loading of therapeutic cargo such as neurotrophic factors (e.g., BDNF, NGF) or anti‐inflammatory RNAs to directly counteract the oxidative stress and apoptosis inherent in neural injury; and second, utilizing neural‐specific ligands (e.g., RVG) for surface modification to achieve a dual‐stage targeting strategy, facilitating both the translocation across biological barriers and subsequent specific neuronal recognition. This dual optimization of barrier penetration and neuro‐repair directly addresses the oxidative stress, neuroinflammation, and BBB restriction that characterize these diseases.

**FIGURE 8:**
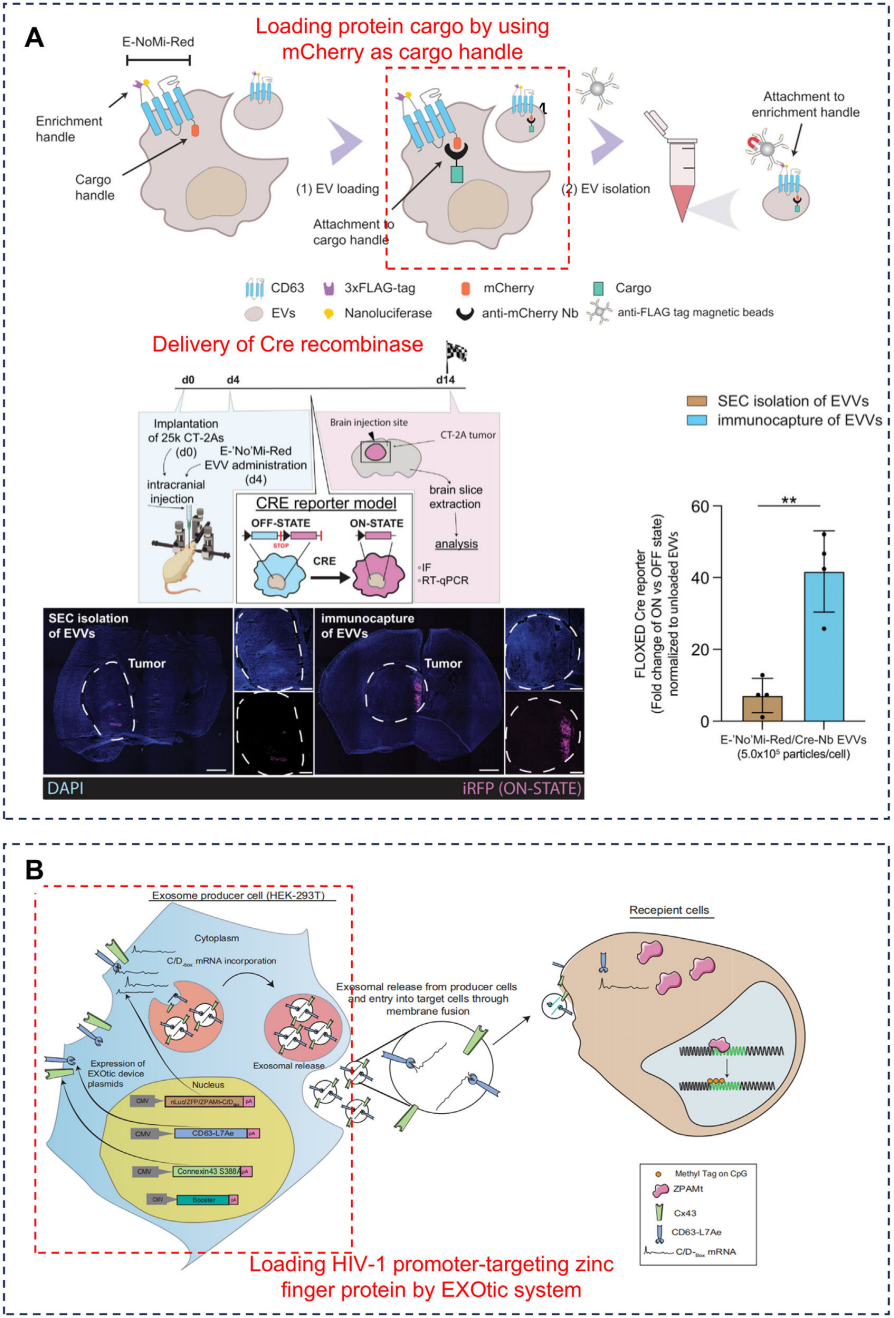
Engineered EVs for therapy of neurological diseases. (A) Schematic of the E‐NoMi system for efficient loading of genome‐editing machinery. The engineered CD63 scaffold (mCherry inside, NanoLuc/FLAG outside) recruits cargo proteins (e.g., Cre, SaCas9) fused to anti‐mCherry nanobodies. These uniformly loaded EVs enable efficient genome editing in the brain, as demonstrated by the Cre‐mediated switch from BFP to IRFP in a reporter system. Reproduced with permission [71]. Copyright 2025, Wiley‐VCH GmbH. (B) In vivo delivery of therapeutic exosomes packaged with the epigenetic repressor ZPAMt reduces HIV‐1 reservoirs in the brain. EVs engineered via the EXOtic system to carry ZPAMt mRNA were administered systemically to HIV‐1‐infected hu‐CD34^+^ NSG mice. Engineered EVs can cross the blood–brain barrier to deliver anti‐HIV‐1 cargo and suppress viral expression in the central nervous system, a key anatomical reservoir for HIV‐1 persistence [124]. Copyright 2024, Springer Nature.

About cargo engineering, modifying parental cells enables the efficient incorporation of therapeutic proteins, RNAs, or gene‐editing tools into EVs. For instance, 3D MSC culture systems have been shown to significantly enhance EV secretion, particularly enriching neurotrophic factors such as BDNF and NGF, which promote neuronal survival and axonal regeneration [108]. This 3D culture environment not only increases the quantity and quality of EVs but also supports their therapeutic potential in counteracting oxidative stress and inflammation. To further optimize this approach, EVs can be loaded with dexamethasone and incorporated into a reactive oxygen species‐responsive hydrogel, enabling sustained release and facilitating functional recovery [101]. To achieve higher precision in protein loading, especially for complex machinery like Cas9 or Cre recombinase, more sophisticated scaffolds have been developed. A prominent example is the E‐NoMi system (EF‐1α‐NanoLuc outside – mCherry inside), where CD63 was re‐engineered with an intracellular mCherry tag to enable selective cargo loading via anti‐mCherry nanobody binding. This system also features an extracellular 3xFLAG tag for immunocapture and enrichment of correctly loaded EVs, which can be removed via a TEV protease cleavage site (Figure 8A) [71]. This dual‐domain design ensured both precise cargo incorporation and efficient downstream isolation, facilitating functional delivery to target cells. The engineered EVs were successfully delivered to the brain via stereotactic intracranial injection. Immunocapture‐derived EVs demonstrated superior cargo loading efficiency and functional delivery compared to SEC‐purified EVs. The Cre‐loxP reporter system confirmed effective genome editing by switching the reporter from BFP (Off‐State) to IRFP (On‐State) upon Cre recombinase delivery. Despite these advances, both strategies have notable limitations. The 3D culture system enhances EV yield and neurotrophic content but lacks precise control over cargo composition. Conversely, the E‐NoMi system achieves high‐precision protein loading (≈55% efficiency) and enrichment (>89% purity) via nanobody affinity and immunocapture, yet it depends on VSV‐G for functional delivery, which raises immunogenicity concerns and lacks tissue‐targeting capability. Moreover, both approaches have been tested primarily via local administration (intracranial or intraspinal injection), and their efficacy via systemic delivery remains unexplored. Future improvements should focus on combining active cargo loading with targeting moieties, eliminating viral fusogens, and validating therapeutic outcomes in clinically relevant disease models.

Beyond serving as highly efficient drug carriers, EVs also hold inherent advantages in targeted delivery, especially in their ability to cross the BBB, which makes them uniquely suitable for neurological disease therapy. Shrivastava et al. utilized this potential to develop an EV‐mediated systemic delivery platform for the stable epigenetic repression of HIV‐1 within the central nervous system. (Figure 8B) [124] By engineering donor cells to endogenously package a chimeric repressor protein comprising an HIV‐1 promoter‐targeting zinc finger protein (ZFP‐362) fused to the active domains of DNA methyltransferase 3A, they produced therapeutic EVs that effectively traversed the BBB. In humanized NSG mouse models, these engineered vesicles successfully delivered their cargo to the brain, where they induced long‐term silencing of latent HIV‐1 through mechanistically driven DNA methylation of the viral promoter, thereby establishing a stable “block and lock” of virus expression in CNS reservoirs. However, the reliance on passive brain accumulation and the potential risk of off‐target DNA methylation still require further optimization before clinical translation.

While EVs possess an intrinsic, albeit limited, ability to cross the BBB, engineering their surface with specific targeting ligands offers a superior strategy for achieving precise cellular specificity and enhanced CNS delivery, particularly for the neuronal transport of siRNA. Building on the natural ability of EVs to cross the BBB, Alvarez‐Erviti et al. developed another strategy to confer precise neuronal specificity [53]. Immunologically inert EVs were derived from immature dendritic cells, which express low levels of MHC‐II and co‐stimulatory molecules such as CD86 to minimize T cell activation. These EVs were engineered by genetically fusing the membrane protein Lamp2b with the RVG peptide, and therapeutic siRNAs were loaded via electroporation. Following systemic administration in murine AD models, the RVG‐modified EVs selectively delivered siRNA to neurons, silencing disease‐related genes and ameliorating amyloid pathology. This pioneering work by Alvarez‑Erviti et al. first demonstrated the feasibility of systemically delivering siRNA to the brain using RVG‑targeted EVs, achieving specific gene knockdown without off‑target effects. However, the electroporation loading efficiency of siRNA was relatively low (<20%), and the reliance on dendritic cells as producer cells limited scalability.

In addition to surface‐ligand engineering for siRNA targeting, bio‐inspired scaffolds provide an alternative approach for orchestrating the assembly and delivery of complex cargos, including mRNA, by leveraging natural physiological recruitment pathways. Genetic modification of parental cells can also enable selective mRNA loading and targeted delivery. For example, EVs engineered with the CD63‐L7Ae RNA‐binding module selectively packaged catalase mRNA containing C/D box motifs, while co‐expression of RVG‐Lamp2b directed EVs to neurons (Figure 5B) [41]. Incorporation of constitutively active Cx43 (S368A) further enhanced cytosolic mRNA delivery. This integrated system achieved efficient catalase mRNA transfer across the BBB, promoted release in CHRNA7‐positive neurons, and reduced oxidative stress and apoptosis in 6‐OHDA‐induced Parkinson's disease models. More advanced design employed endogenous retrovirus‐like scaffolds such as Arc to create engineered Arc‐EVs (eraEVs), taking advantage of Arc's homology to retroviral Gag proteins, which naturally bind and encapsulate mRNAs. The Arc capsid self‐assembles within leukocyte‐derived EVs, and the inclusion of the Arc 5′ untranslated region (A5U) further stabilizes the cargo and enhances packaging efficiency. Leukocyte‐derived EVs were chosen because they can cross the BBB during neuroinflammation and carry adhesion molecules that promote accumulation in inflamed neuronal regions. Within parental leukocytes, Arc complexes containing A5U RNA and therapeutic mRNAs assemble and are secreted as eraEVs [43], which, upon systemic administration, were internalized by neurons. The delivered mRNAs are efficiently translated, leading to suppression of neuroinflammatory pathways, reduced microglial activation, and improved neuronal function in aged mice. While this work highlights the potential of eraEVs as a neuron‐targeted mRNA delivery platform, further studies are needed to explore loading of other RNA types, in vivo production by engineered parental cells, and additional membrane modifications for selective targeting. While these bio‑inspired systems represent significant advances in mRNA packaging and neuronal delivery, both strategies face common translational hurdles. The EXOtic system relies on transient transfection of parental cells and the implantation of live cells, complicating scale‑up and raising safety concerns. The eraEV platform, despite its improved cargo loading via Arc capsids, still depends on primary leukocyte cultures and requires separation of the stabilizing A5U element from the therapeutic mRNA, adding production complexity. Moreover, neither system has yet demonstrated therapeutic efficacy in clinically relevant disease models beyond proof‑of‑concept studies. Future refinements should focus on establishing stable producer cell lines, simplifying vector design, and validating functional outcomes in chronic neurodegenerative disease models to facilitate clinical translation.

Collectively, these approaches demonstrated how molecular and genetic manipulation of parental cells can produce EVs capable of combating oxidative injury, neuroinflammation, and neuronal loss. The evolution of EV targeting strategies is evident, progressing from the modulation of the BBB to the selective delivery of therapeutic molecules to neurons, and ultimately advancing to targeted gene therapy. This progression highlights how rational engineering of EV composition and surface properties enables more precise, effective, and comprehensive treatments for neurological diseases.

### 4.4 Cancer

Cancer represents a major global health burden, accounting for a significant proportion of both morbidity and mortality worldwide. Tumor development is driven by a complex interplay between malignant cells and their surrounding microenvironment. Oncogenic mutations, such as those in KRAS [125], promote uncontrolled proliferation and metabolic reprogramming, while cancer‐associated fibroblasts (CAFs), MDSCs [68], regulatory T cells (Tregs) [102], and other immunosuppressive cells secrete cytokines that dampen cytotoxic T‐cell activity and promote immune evasion. Excessive deposition of extracellular matrix components, including hyaluronan (HA), collagens, fibronectin, and versican, etc., forms a dense stromal barrier that restricts immune cell infiltration and drug penetration [104]. Concurrently, overexpression of immune checkpoint molecules like PD‐L1 on tumor and stromal cells inhibits T‐cell activation, fostering immune escape [106]. Collectively, these alterations give rise to an immune‐excluded and therapy‐resistant tumor microenvironment that enables tumor maintenance and limits therapeutic efficacy. Current chemotherapy faces significant limitations, including nonspecific drug distribution and limited therapeutic responses, and they often fail to achieve durable remission in advanced or metastatic cancers. In recent years, biological therapeutics‐including immune‐based treatments, protein and antibody drugs, cytokine therapies, and emerging gene and enzyme modalities‐have advanced rapidly, with an increasing emphasis on precise targeting, controlled biodistribution, and multimodal synergy to address tumor heterogeneity and remodel pathological microenvironments [126]. In cancer therapy, the engineering application of EVs focuses on the precise targeting of tumor cells and the modulation of immune cells, designing them as “immunoregulatory hubs.” The core of this strategy lies in utilizing ligands such as scFv and Z‐domain not only to achieve high‐precision recognition of tumor cells, but also to directly target or even functionally convert key immunosuppressive populations in the tumor microenvironment, such as MDSCs and CAFs. This strategic transition from “killing single cells” to “remodeling the entire tumor niche” is precisely the core characteristic of cancer EV engineering.

**FIGURE 9:**
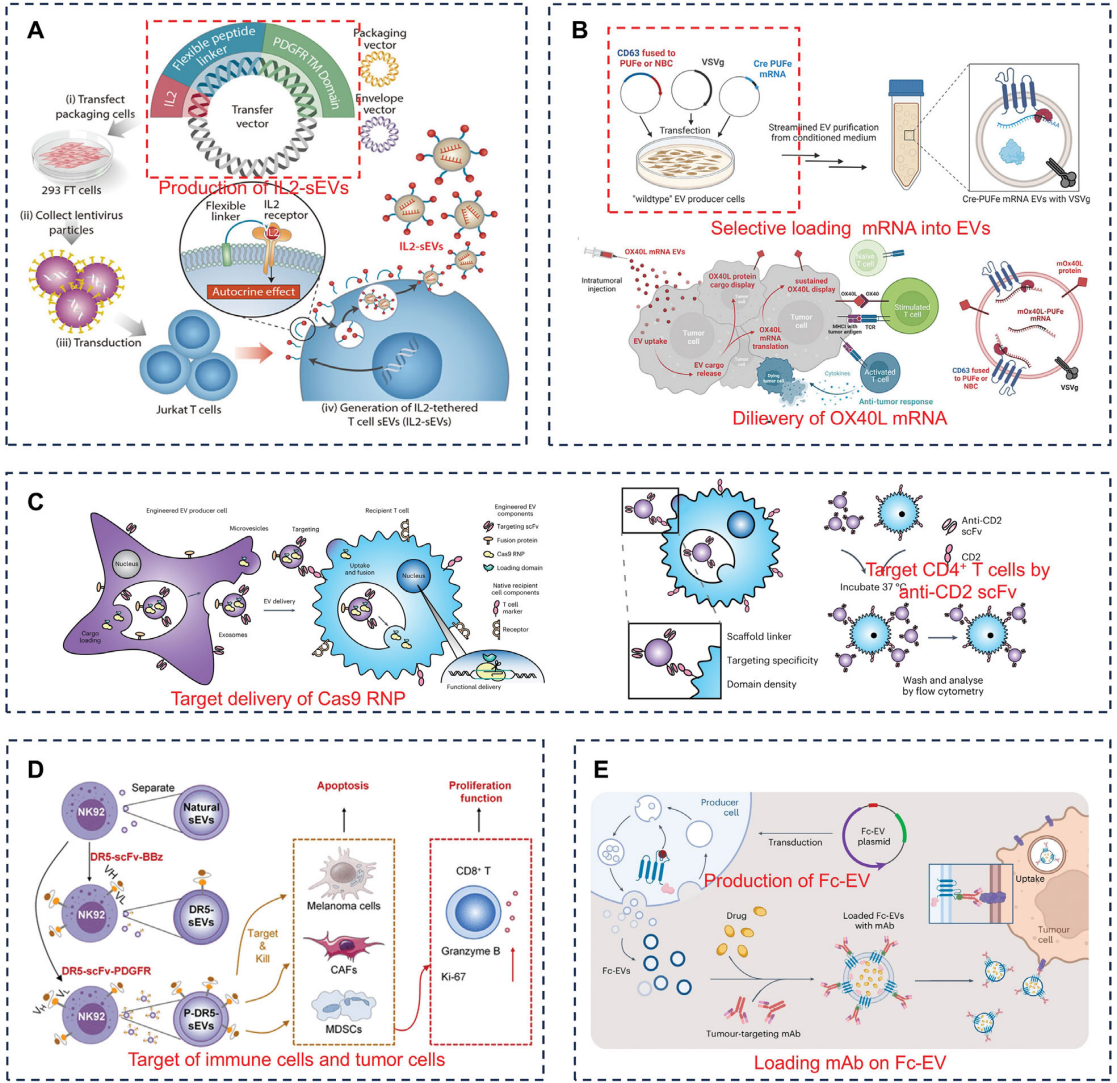
Engineered EVs for enhanced cancer immunotherapy. (A) Schematic of IL2‐sEVs generated by lentivirally engineered Jurkat T cells expressing membrane‐anchored IL‐2 via fusion to the PDGFR transmembrane domain. The surface‐tethered IL‐2 creates an autocrine activation loop that enhances T‐cell stimulation and drives the release of IL2‐sEVs enriched in immunomodulatory miRNAs, collectively strengthening antitumor immune responses. Reproduced with permission [102]. Copyright 2022, Wiley‐VCH GmbH. (B) Multi‐stage engineered EVs for efficient mRNA delivery. The CD63‐PUFe fusion enables selective mRNA packaging, while VSV‐G enhances endosomal escape. Delivery of OX40L mRNA provides sustained T cell co‐stimulation, inducing potent antitumor immunity. Reproduced with permission [42]. Copyright 2024, Wiley‐VCH GmbH. (C) Schematic of CRISPR/Cas9‐loaded EVs for T cell engineering. Surface‐displayed anti‐CD2 scFv enables T cell targeting, and the ABA‐inducible ABI‐PYL system facilitates specific loading of Cas9/sgRNA complexes to disrupt genes like CXCR4 in primary T cells. Reproduced with permission [46]. Copyright 2021, Wiley‐VCH GmbH. (D) Schematic of engineering natural killer cell‐derived EVs (NK‐EVs) displaying a DR5‐agonistic scFv. These EVs induce apoptosis in DR5^+^ tumor cells, MDSCs, and CAFs, while activating cytotoxic T lymphocytes for robust tumor regression. Reproduced with permission [68]. Copyright 2025, Elsevier. (E) Modular Fc‐EV platform for flexible antibody‐guided targeting. The CD63‐Z‐domain fusion allows high‐density loading of various IgGs (e.g., anti‐PD‐L1, anti‐HER2). When co‐loaded with drugs like doxorubicin, these EVs achieve highly specific tumor targeting and potent therapeutic efficacy. Reproduced with permission [103]. Copyright 2024, Springer Nature.

To address these multifaceted pathological barriers, current cargo engineering efforts in cancer therapy primarily prioritize the loading of therapeutic proteins as direct and immediate executors of functional remodeling. Due to their inherent immune‐modulatory properties and preferential interactions with other immune and cancer cells, T cell‐derived EVs provide a natural platform for anticancer therapy. Jung et al. used lentiviral transduction to engineer Jurkat T cells to express interleukin‐2 (IL‐2) fused via a flexible linker to the PDGFR transmembrane domain, enabling an autocrine loop that enhanced IL‐2 signaling and led to the secretion of IL‐2‐decorated small EVs (IL2‐sEVs) (Figure 9A) [102]. These IL2‐sEVs functioned as potent immunostimulatory platforms that effectively activated CD8^+^ T cells to mount a robust response against melanoma. While the therapeutic effect was primarily driven by the surface‐anchored IL‐2, the autocrine signaling within the parental Jurkat cells also led to the enrichment of specific miRNAs (such as miR‐181a‐3p and miR‐223‐3p) in the sEVs, which further sensitized melanoma cells to cytotoxicity by downregulating PD‐L1. In immunocompetent melanoma‐bearing mice, IL2‐sEVs significantly inhibited tumor progression and synergized with chemotherapeutic or immune checkpoint agents to further enhance anticancer efficacy. Despite these promising outcomes, IL2‐sEVs lack intrinsic tumor‐specific targeting, and their distribution to nontumor tissues may limit precision, indicating a need for additional strategies to improve selective delivery in clinical applications. In another strategy, researchers anchored PH20 to HEK293T EVs membranes via a GPI anchor, constructing Exo‐PH20 that degrades HA in the tumor matrix to facilitate drug penetration and T‐cell infiltration. Exo‐PH20 efficiently degraded HA, improving doxorubicin delivery and immune cell infiltration. In vivo, Exo‐PH20 enhanced doxorubicin distribution and promoted CD8^+^ T‐cell infiltration, leading to stronger tumor suppression compared to free drug treatment. Collectively, Exo‑PH20 exhibits potent efficacy in degrading HA and improving chemotherapy distribution, achieves high enzymatic activity at a low dose (≈53 µg/kg PH20), and avoids the thromboembolic risks associated with high‑dose PEGPH20. Yet, its dependence on EPR‑mediated passive accumulation and HEK293T‑derived exosome safety calls for active targeting strategies and rigorous product characterization before clinical translation [104].

In alignment with the aforementioned strategies for protein cargo loading, Zickler et al. developed a multistage EV engineering platform (Figure 9B) [42]. Utilizing the previously discussed modular loading mechanism involving CD63 fused with the high‐affinity RNA‐binding domain PUFe, which recognizes an engineered 8‐nucleotide motif in the 3′UTR of target transcripts, the researchers achieved the selective and high‐efficiency packaging of OX40L mRNA into HEK293T‐derived EVs. To overcome the subsequent challenge of endosomal entrapment, the fusogenic protein VSV‐G was integrated into the EV membranes to promote endosomal escape and enhance cytosolic release upon uptake. VSV‐G mediates host‐cell entry through low‐pH‐induced conformational rearrangements that expose a hydrophobic fusion loop, thereby driving membrane merger between viral and endosomal membranes [72]. The EVs not only provided rapid activation of T cells via surface OX40L protein but also sustained immune stimulation through translated OX40L mRNA, collectively reprogramming the tumor immune milieu. In a B16F10 melanoma model, this strategy achieved up to 67% complete tumor remission and prolonged survival without detectable systemic toxicity, demonstrating potent and durable antitumor immunity at doses orders of magnitude lower than conventional LNP‐based formulations. This endogenous platform achieves highly efficient mRNA loading with over 230fold enrichment and, via VSV‐G, enables functional delivery at subng/kg doses, orders of magnitude lower than LNPs. However, it relies on HEK293T cells and a viral fusogen, posing immunogenicity risks, and systemic efficacy remains unproven. Future optimisation should focus on alternative fusogens, scalable production, and active targeting to reduce off‐target effects.

Beyond the delivery of individual proteins or nucleic acids, the engineering of EVs for the transport of functional protein–nucleic acid complexes represents a critical progression toward sophisticated therapeutic applications such as gene editing. For instance, Devin et al. employed HEK293FT cells as the producer EV‐producing platform, leveraging their high EV yield and robust transfection efficiency (Figure 9C) [46]. The engineering strategy involved lentiviral transduction of the parental cells with constructs encoding anti‐CD2 single‐chain variable fragments (scFvs) fused to the PDGFR transmembrane domain for stable surface display, while Cas9 was C‐terminally tagged with the ABI domain to enable selective loading into EVs via the abscisic acid (ABA)‐inducible ABI‐PYL dimerization system. Consistent with the aforementioned use of VSV‐G to facilitate endosomal escape, this platform integrated specialized fusogenic modules to ensure efficient cytosolic delivery of the gene‐editing machinery. In addition to the broad membrane‐fusion capability of VSV‐G, measles virus H/F glycoproteins were incorporated to achieve both specific T‐cell targeting and receptor‐mediated fusion, thereby enhancing the precision of the delivery vehicle. In vitro studies demonstrated that these multifunctional EVs efficiently delivered Cas9/sgRNA complexes into primary human CD4^+^ T cells, achieving site‐specific disruption of the CXCR4 locus with indel frequencies up to ∼1.3% for VSV‐G decorated exosomes, and repeated dosing further increased editing rates to ∼2.8%. Although these editing efficiencies remain below those achieved by standard Cas9 RNP electroporation, the EV‐based approach offers noninvasive, receptor‐targeted delivery with minimal cytotoxicity. While the system provides a versatile and modular framework for intracellular cargo delivery, its efficacy in vivo and therapeutic relevance in disease models remain to be further validated.

Another major research direction involves tumor homing or condition‐adapted targeting, where the targeting mechanism is often driven by the tumor microenvironment or the cellular origin of the EVs. For instance, macrophage‐tumor chimeric exosomes (aMT‐exos), which integrate the antigen‐presenting properties of M1 macrophages with the intrinsic tumor‐homing capacity of cancer cells, have been shown to accumulate within both lymph nodes and tumor tissues [127]. These hybrid EVs effectively activate antigen‐specific T‐cell responses and reprogram the immunosuppressive tumor microenvironment, resulting in marked inhibition of primary, metastatic, and recurrent tumor growth. In another study, tumor cells such as MGC803 and HepG2 were cultured under acidic conditions (pH 4.0) to produce exosomes (LP‐Exos) with altered lipid composition, enhancing membrane fusion and tumor cell uptake [105]. These LP‐Exos were engineered with homotypic targeting for improved tumor homing and dual‐loaded with aluminum phthalocyanine (AlP) and Dox. Upon near‐infrared (NIR) light activation, AlP generated reactive oxygen species (ROS), disrupting the exosome membrane and releasing Dox, thereby enhancing antitumor efficacy through photodynamic therapy and chemotherapy, with minimal toxicity.

A major advancement in targeted therapy is the shift from broad targeting approaches to precise ligand‐mediated targeting, where EVs are engineered to carry specific binding domains that selectively recognize tumor‐associated surface markers. Leveraging the innate cytotoxic machinery, low immunogenicity, and effector molecule‐carrying capacity of NK cells, Guo et al. engineered NK92 cells via lentiviral transduction to express a DR5‐agonistic scFv fused to the PDGFR transmembrane domain, removing CAR signaling motifs to focus on apoptosis induction (Figure 9D) [68]. The resulting natural killer cell‐derived small extracellular vesicles (NK‐sEVs) displayed DR5‐scFv on their surface, exploiting the preferential overexpression of DR5 on multiple cancer types, including melanoma, liver, and breast cancers, as well as on immunosuppressive populations within the tumor microenvironment, such as MDSCs and CAFs. Functionally, DR5‐scFv sEVs rapidly induced apoptosis of DR5^+^ cancer cells, MDSCs, and CAFs in vitro and specifically accumulated in DR5^+^ tumors in vivo. Systemic administration significantly inhibited tumor growth in melanoma, liver, and breast cancer models, prolonged survival in mice, and outperformed conventional DR5 antibodies. In organotypic patient‐derived melanoma slice cultures, DR5‐scFv sEVs effectively suppressed tumor cells and MDSCs while activating antitumor immune responses, demonstrating their capacity to remodel the tumor microenvironment and enhance therapeutic efficacy. A related strategy employed bispecific dendritic cell‐derived EVs displaying an anti‐CD19 scFv anchored via the hinge‐transmembrane domain and PD‐1 incorporated through its natural transmembrane domain [106]. These EVs selectively bound CD19^+^ tumor cells while blocking PD‐L1‐mediated immunosuppression, enhancing tumor accumulation and efficacy. The dendritic cell origin also contributed MHC peptides and co‐stimulatory molecules (CD80/86), reducing suppressive populations such as Tregs and MDSCs, resulting in significant tumor growth inhibition and prevention of metastasis. Building on conventional antibody‐coupling strategies, a more advanced approach involved engineering EVs with a CD63‐Z‐domain Fc‐binding module (Fc‐EVs), enabling flexible, high‐density attachment of virtually any IgG antibody and greatly expanding the adaptability of antibody‐guided EV targeting (Figure 9E) [103]. When functionalized with PD‐L1 or HER2 antibodies and co‐loaded with Dox, these Fc‐EVs showed exceptional targeting performance, increasing uptake by PD‐L1^+^ or HER2^+^ tumor cells by more than 300–500‐fold in vitro and achieving up to 7.25% ID/g tumor accumulation in vivo. This antibody‐guided delivery markedly enhanced tumor‐specific cytotoxicity, improved intratumoral drug retention, and produced strong therapeutic benefit, including complete survival in treated mice. The modular Fc‐EV architecture offers clear advantages in design versatility, enhanced functional performance, and seamless compatibility with a broad range of antibody modalities; however, its reliance on exogenous antibodies may increase production complexity, and future optimization could focus on scalable antibody‐EV assembly and integrating multispecific antibody formats to further refine targeting precision. Nevertheless, these targeted delivery strategies are still hindered by common hurdles, most notably the risk of insertional mutagenesis associated with their dependence on lentiviral transduction. The selected antigens (DR5, CD19, PDL1/HER2) are also present on normal tissues, posing risks of ontarget offtumor toxicity. Furthermore, low EV yields from parental cells (primary dendritic cells or NK92 cells) and complex purification processes hinder scalable manufacturing. Future improvements should prioritize nonviral engineering methods, tumorspecific or dualtargeting moieties to reduce offtumor effects, and robust bioprocessing technologies. Collectively, these studies illustrate a progressive evolution from environment‐driven or homotypic targeting to precise ligand‐guided engineering, demonstrating how rationally designed EVs can achieve both specific tumor recognition and immune modulation, thereby advancing their translational potential in cancer therapy.

## 5 Conclusion and Future Perspective

EVs have emerged as promising therapeutic agents due to their natural enrichment of bioactive molecules and their ability to inherit functional properties from parental cells. This inherent potential is further expanded by engineering strategies that enable efficient cargo loading and target modification, thereby transforming EVs into programmable therapeutic nanocarriers. In this review, we have summarized the mechanisms of EV biogenesis and natural molecular sorting, highlighted endogenous engineering strategies for protein and nucleic acid loading, and discussed their broad applications in cardiovascular repair, liver protection, neurological disorders, and cancer therapy.

Scaffold proteins represent essential tools for directing therapeutic proteins or nucleic acids into EVs. Recent advances in high‐throughput analysis and single‐vesicle quantification have greatly deepened our understanding of how EV cargos are selected and packaged. Systematic proteomic studies‐such as those identifying TSPAN14, PTGFRN, BASP1, and PLXNA1 as highly efficient scaffold proteins‐have generated valuable datasets linking protein sequence, structure, and loading performance. Integrating these data with artificial intelligence (AI) could shift EV bioengineering from empirical trial‐and‐error toward a predictive and data‐guided design process. By building training sets that include features such as amino acid sequence, membrane topology, localization motifs, and measured loading efficiency, machine‐learning models like graph neural networks or multitask frameworks could predict new scaffold candidates and their optimal fusion orientations before experimental testing. In the case of RNA sorting, Garcia‐Martin et al. identified hundreds of EV‐enriched (EXOmotifs) and cell‐retained (CELLmotifs) sequences across multiple metabolically active cell types [17]. Given that thousands of possible short RNA motifs remain unexplored, AI models trained on experimentally validated motifs could provide meaningful guidance for pinpointing additional sequences that enhance EV loading. More broadly, this computational approach offers a new perspective for discovering combinatorial enrichment codes that are difficult to capture using traditional statistical analyses. For protein‐based scaffolds, however, the search space is far larger because amino acid sequences are virtually unlimited. Here, AI prediction must rely on biological constraints‐such as membrane localization, transmembrane domain architecture, and evolutionary conservation‐to focus on plausible EV‐sorting candidates. By continuously learning from quantitative datasets and iterative experimental feedback, AI‐driven modeling could accelerate the identification of both sorting motifs and scaffolds that define EV cargo selectivity. Ultimately, combining high‐throughput screening, quantitative single‐vesicle analysis, and AI will enable a new generation of programmable EVs with predictable composition, controllable functionality, and improved therapeutic performance.

While the field has progressed, the clinical translation of engineered EVs is currently restricted by a series of technical and biological barriers. First, even when target proteins are successfully loaded via scaffold fusions or tag interaction, their functional delivery may be compromised if the cargo fails to dissociate from its membrane anchor upon arrival in recipient cells, thereby limiting the intended bioactivity. Second, following cellular uptake, the majority of internalized EVs remain entrapped within endosomal compartments, and inefficient endosomal escape continues to represent a universal rate‑limiting step that restricts cytosolic access of the therapeutic cargo. Finally, large‐scale production of endogenously engineered EVs introduces unique challenges in quality control, as the stability of genetic modifications at the parental cell level and the consistency of cargo loading efficiency and EV‐level cargo heterogeneity across production batches can vary over time, thereby complicating standardization and reproducibility.

Future progress will depend on innovations across multiple fronts. To address the issue of incomplete cargo release, controlled release strategies using optogenetic cleavage or pH‐sensitive inteins offer a means to deposit free cargo into the EV lumen. Similarly, tethering strategies based on lipidation or peptide tags provide an alternative loading route. Regarding the persistent barrier of endosomal entrapment, the incorporation of viral fusogens such as VSV‑G markedly improves endosomal escape, and ongoing efforts to mitigate the immunogenicity of viral components through protein engineering or the exploration of alternative fusogenic proteins will be valuable for advancing this approach. Beyond these delivery‐focused innovations, manufacturing scale‑up constitutes an equally critical frontier. Finally, to support clinical translation, robust quality control frameworks tailored to endogenous engineering will be required, including real‐time sampling to monitor the stability of genetic modifications in parental cells, as well as batch‐to‐batch consistency of key engineered features at both the cellular and EV levels. Ultimately, a concerted effort that integrates cell‑engineering precision, refined delivery‑release mechanisms, and robust manufacturing pipelines will be essential to transform endogenously engineered EVs into reliable, off‑the‑shelf therapeutic products. With these orchestrated innovations, engineered EVs are poised to evolve from experimental constructs into standardized, programmable therapeutics, offering a powerful new paradigm for the next generation of precision medicine.

## Conflicts of Interest

The authors declare no conflicts of interest.

## Data Availability

The authors have nothing to report.
